# Design and Validation of Linkers for Site-Specific Preparation of Antibody–Drug Conjugates Carrying Multiple Drug Copies Per Cysteine Conjugation Site

**DOI:** 10.3390/ijms21186882

**Published:** 2020-09-19

**Authors:** Amit Kumar, Shenlan Mao, Nazzareno Dimasi, Changshou Gao

**Affiliations:** 1 Antibody Discovery and Protein Engineering Department, AstraZeneca R&D, Gaithersburg, MD 20878, USA; amit.kumar@astrazeneca.com (A.K.); DimasiN@vielabio.com (N.D.); 2 AstraZeneca Oncology R&D, Gaithersburg, MD 20878, USA; shenlan.mao@astrazeneca.com

**Keywords:** antibody–drug conjugate (ADC), drug to antibody ratio (DAR), heterofunctional linker, site-specific conjugation, cysteine conjugation, branched linker, high-DAR ADC

## Abstract

First-generation cysteine-based site-specific antibody–drug conjugates (ADCs) are limited to one drug per cysteine. However, certain applications require a high drug to antibody ratio (DAR), such as when low-potency payloads are used. Higher drug load can be achieved using classical cysteine conjugation methods, but these result in heterogeneity, suboptimal efficacy and pharmacokinetics. Here, we describe the design, synthesis and validation of heterobifunctional linkers that can be used for the preparation of ADCs with a DAR of two, three and four in a site-specific manner per single cysteine conjugation site, resulting in site-specific ADCs with a DAR of four, six and eight. The designed linkers carry a sulfhydryl-specific iodoacetyl reactive group, and multiple cyclic diene moieties which can efficiently react with maleimide-carrying payloads through the Diels–Alder reaction. As a proof of concept, we synthesized site-specific DAR four, six and eight ADCs carrying tubulysin (AZ13601508) using engineered antibodies with a cysteine inserted after position 239 in the antibody CH2 domain. We evaluated and compared the in vitro cytotoxicity of ADCs obtained via the site-specific platform described herein, with ADCs prepared using classical cysteine conjugation. Our data validated a novel cysteine-based conjugation platform for the preparation of site-specific ADCs with high drug load for therapeutic applications.

## 1. Introduction

Antibody–drug conjugates (ADCs) are a class of therapeutic agents that are comprised of a monoclonal antibody tethered to a potent cytotoxic molecule through a chemical linker. The Food and Drug Administration (FDA) approval of several ADCs has provided tremendous impetus to research in this field. Most ADCs approved or in clinical development employ traditional conjugation methods that are either lysine–amide coupling or cysteine–maleimide conjugation to tether the cytotoxic molecule to the antibody. These methods, though simple to use, produce heterogeneous conjugates complicating analytical characterization and batch reproducibility. The cysteine–maleimide conjugation strategy faces an additional problem of serum instability [1]. To address the shortcomings of classical conjugation, the ADC field is gradually moving towards site-specific conjugation approaches. The site-specific approaches improve the homogeneity of ADC products, the manufacturability and the ease of characterization [2,3]. Further, in certain cases, site-specific conjugates have been reported to show improved efficacy, stability and safety [4,5,6].

To date, numerous site-specific conjugation approaches have been developed, each with their own advantages and disadvantages. Despite the availability of a plethora of site-specific conjugation approaches, most methods developed to date lead to a drug to antibody ratio (DAR) of only two. Branched linkers based on site-specific conjugation approaches may be used for loading multiple payload molecules onto an antibody but have not been fully explored. These linkers may lead to site-specific high-DAR antibody–drug conjugates with minimal disruption to the antibody structure and may even enhance ADC efficacy. To date, only a handful of branched linkers have been described in the literature that could lead to a high DAR in a site-specific manner. Anamiet et al. developed branched linkers utilizing microbial transglutaminase-mediated site-specific conjugation to provide a platform for attaining a DAR of 4 [7]. Chen et al. and Levengood et al. also reported linkers that carry two drug units per linker that were used for classical hinge cysteine-based conjugation [8,9].

**Figure 1:**
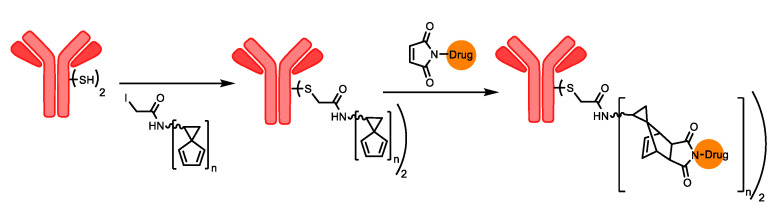
Schematic representation of the strategy for attaining high-drug to antibody ratio (DAR) antibody–drug conjugates (ADCs) in a site-specific manner.

In this work, we describe branched linkers that can be used for the preparation of ADCs with a DAR of four, six and eight in a site-specific manner. The designed linkers carry a sulfhydryl-specific iodoacetyl reactive group and can be utilized for making high-DAR ADCs in a site-specific manner using cysteine engineered antibodies (Figure 1). To demonstrate the effectiveness of our platform, we use well-validated antibodies with cysteine insertion after position 239 (EU numbering) in the CH2 domain of the antibody heavy chain sequence to make a platform for achieving DARs of four, six and eight in a site-specific manner using a two-step procedure. [10,11] The described branched linkers carry multiple cyclic diene moieties which can efficiently react with maleimide-carrying payloads through the Diels–Alder reaction (Figure 1). The linker antibody platform is modular in nature and can be used for attaching any maleimide (most commonly found functionality among the payloads for cysteine conjugations) carrying payload to yield a high drug load in a site-specific manner.

## 2. Results and Discussion

**Figure 2:**
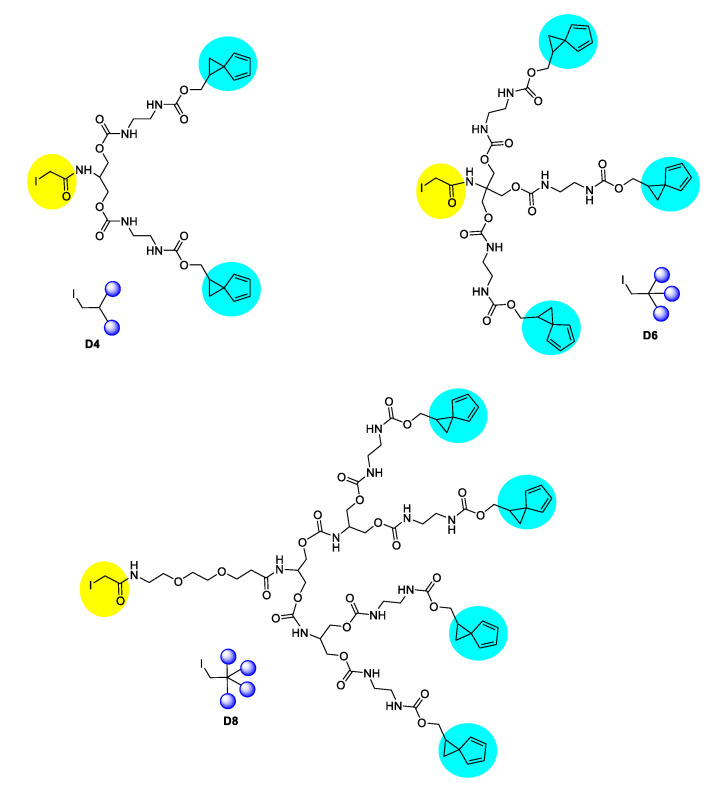
Design of the branched linkers used for preparing high-DAR ADCs in a site-specific manner.

Cysteine residues of monoclonal antibodies remain a popular site for conjugation and are most often targeted by maleimide-based payloads. However, the newly formed thiosuccinimide ring resulting from thiol–maleimide chemistry is susceptible to thiol exchange or retro-Michael addition, resulting in premature drug release leading to lower efficacy and increased off-target toxicity [12]. One way to overcome the instability of thiol–maleimide conjugation is to use alternative conjugation methods leading to plasma stable thioethers at cysteine in a rapid and efficient manner. The reaction of thiol with the iodoacetyl group to form a stable thioether bond is one such conjugation technique and has been previously employed for making ADCs [12,13,14,15]. The design of branched heterofunctional linkers for obtaining high-DAR ADCs is shown in Figure 2. Each of the described branched linkers uses the iodoacetyl reacting group to conjugate cysteine-inserted antibodies in an efficient manner at elevated pH to form a stable thioether linkage. The linkers carry cyclic dienes which react efficiently via the Diels–Alder reaction with commonly available maleimide payloads to form plasma stable ADCs [16]. Our DAR 4 linker (D4) carries two cyclic dienes capable of reacting with two maleimide payloads, while our DAR 6 linker (D6) carries three and DAR 8 linker (D8) carries four cyclic diene units. While each of our D4 and D6 linkers carry a short spacer to conjugate free cysteines in an antibody, our D8 linker carries a diethylene glycol unit (PEG 2) to overcome any potential steric hinderance caused by the bulky nature of the linker.

**Scheme 1:**
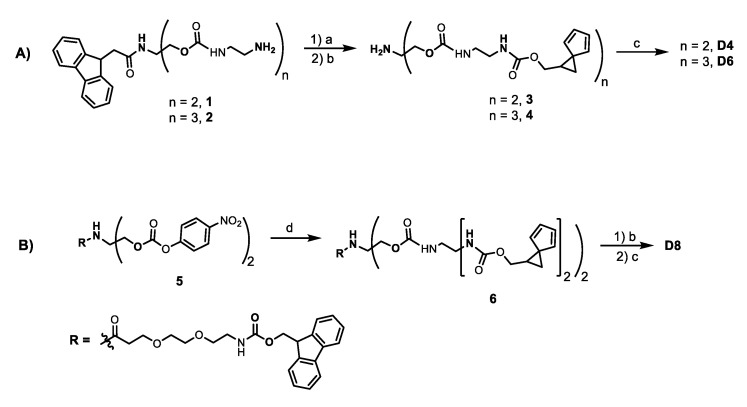
Synthesis of (**A**) D4, D6 and (**B**) D8 linkers. (a) 4-nitrophenyl (spiro[2,4]hepta-4,6-dien-1-ylmethyl) carbonate, *N*,*N*-Diisopropylethylamine (DIPEA), (b) 50% Dimethylamine in Dimethylformamide, (c) Iodoacetic anhydride, (d) compound 3, DIPEA.

**Figure 3:**
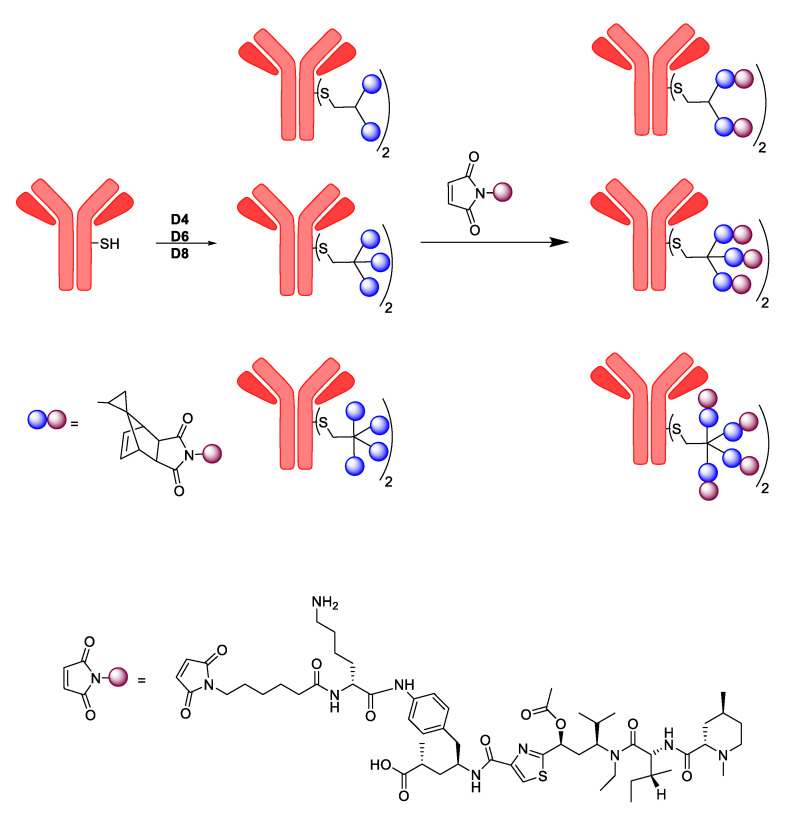
Preparation of DAR 4, 6 and 8 ADCs in a site-specific manner.

The synthesis of the designed branched linkers is described in Scheme 1 (detailed procedure for the intermediates can be found in the Supplementary Information). The synthesis of the D4 and D8 linkers used commercially available serinol, while the D6 linker started from commercially available tris(hydroxymethyl)aminomethane (tris base). Serinol and tris base were converted to compounds 1 and 2, respectively, in four steps and the procedure is fully described in the Supplementary Information. Fluorenylmethyloxycarbonyl (Fmoc)-protected compounds 1 and 2 were converted to free amine using a 50% mixture of dimethylamine (DEA) in dimethylformamide (DMF) (Scheme 1A). DEA was preferred over 4-methyl piperidine for the deprotection of Fmoc as the dibenzofulveve-piperidine adduct, formed from the deprotection step, overlapped with the desired products (3 and 4) during chromatographic separation. The DEA adduct was found to be much more polar and easier to separate. The amine precursor of D4 and D6 was converted to the desired final product using iodoacetic anhydride under basic conditions. As mentioned before, synthesis of the D8 linker started with the reaction of tris base with Fmoc-PEG2-NHS followed by activation of hydroxy groups with 4-nitrophenyl chloroformate to give compound 5 (Supplementary Information). The activated hydroxyls of 5 were reacted with compound 3 (D4 precursor) to give Fmoc-protected 6 carrying four diene units. The Fmoc protecting group was removed from 6 using a 50% mixture of DEA in DMF to give the D8 precursor which was reacted with using iodoacetic anhydride under basic conditions to yield the desired product drug loading onto an antibody is shown in Figure 3. In the first step, the synthesized linkers were conjugated to antibodies engineered to carry a free cysteine inserted after position 239. To demonstrate a proof of concept, trastuzumab, an anti-HER2 antibody, engineered to carry a free cysteine inserted after position 239 was used as a positive control. NIP228 also carrying inserted cysteine after position 239 was used as an isotype control. For the conjugation of the branched linkers, the cysteine-inserted antibodies were first processed to free up any disulfide bonds associated with inserted cysteines. To do this, engineered antibodies were incubated at 37 °C with (tris(2-carboxyethyl)phosphine) (TCEP) to reduce all the solvent accessible disulfides (detailed protocol can be found in the Materials and Method section). The resulting sample was dialyzed and then oxidized with dehydroascorbic acid to reform the antibody interchain disulfide bonds. This process resulted in two conjugation ready free cysteines per antibody (i.e., the inserted cysteines after position 239). The conjugation of the engineered antibody with branched linkers was carried out at elevated pH (8–8.5) resulting in antibody–linker conjugate that could be used for generating high-DAR ADCs. It should be noted that the iodoacetyl-based conjugation with a cysteine-inserted antibody is thiol-specific at the specified pH range [14,15]. The antibody concentration employed for conjugating branched linkers ranged from 2 to 4 mg/mL (Supplementary Information). The D4 and D6 linkers used eight equivalents of the linker (per cysteine) with a reaction time of 1.5 h. The D8 linker, being bulkier, employed 12 equivalents (per cysteine) with a reaction time of 3 h (detailed protocol can be found in the Materials and Method section). Ceramic hydroxyapatite chromatography (CHT) was used to remove the unreacted linkers and the macromolecular aggregates that were formed during conjugation. The CHT-purified linker–antibody conjugates were determined to be over 95% monomer by size-exclusion chromatography (SEC-HPLC, data not shown). Conjugation efficiencies for the conjugation of branched linkers with cysteine engineered antibodies was determined using reduced liquid chromatography-mass spectrometry (LC-MS) and showed a conjugation efficiency of over 90% for all the linkers (detailed protein mass spectrum available in the Supplementary Information). LC-MS results also showed conjugation on the heavy chain only which confirmed that the conjugation was specific to the antibody heavy chain where the inserted cysteine was introduced. The reaction rate and conjugation efficiencies for the reaction between linkers with cysteine-inserted trastuzumab and NIP228 were found to be similar (Supplementary Information).

In the second step of the two-step conjugation method, the antibody–linker conjugates were reacted with a maleimide payload. We chose AZ13601508 (AZ-1508), a tubulysin warhead and maleimide-based linker for demonstrating the proof of concept (Figure 3) [17]. Our antibody–linker conjugates carry multiple diene units designed to conjugate maleimide payloads via the Diels–Alder reaction at a broad pH rage [16]. Antibody–linker conjugates were incubated with AZ-1508 payload to convert them into ADCs. As maleimides at high concentrations have a propensity to target hinge disulfides of an antibody at elevated pH (pH ≥ 7.2), and to prevent their hydrolysis, the conjugation of the payload with antibody–linker conjugates was performed at lower pH (pH~6.0). Antibody conjugates of the D4, D6 and D8 linkers used 8, 12 and 16 equivalents of AZ-1508, respectively, to complete the reaction in 3 h. Purification of the conjugation mixture was performed by CHT and the poled fraction was determined to be over 95% monomer by size-exclusion chromatography (Supplementary Information). Conjugation efficiency of the CHT-purified ADCs was determined to be greater than 95% by LC-MS. The results also showed addition of AZ-1508 on the heavy chain, while the light chain remained unaffected (detailed protein mass spectrum available in the Supplementary Information). No difference in rate or conjugation efficiencies for the reaction between antibody–linker conjugates and AZ-1508 was detected.

**Figure 4:**
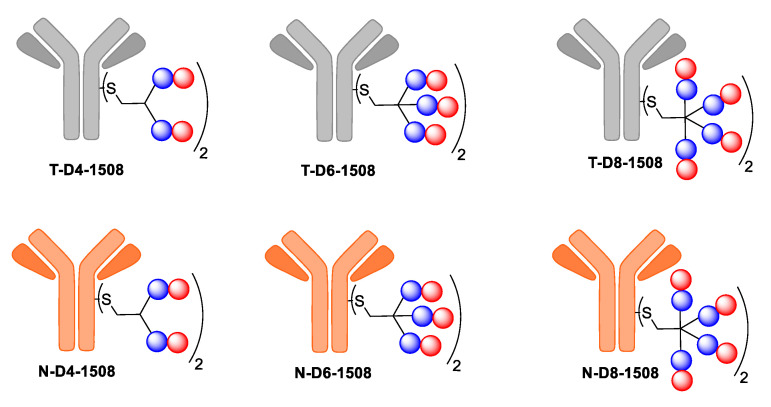
Schematic representation of trastuzumab- and NIP228-derived antibody–drug conjugates synthesized using linkers 1, 2 and 3 and cytotoxic payload AZ-1508.

As described in Figure 3, a two-step conjugation approach was used for the preparation of DAR4, DAR6 and DAR8 ADCs using trastuzumab, an anti-HER2 antibody, and NIP228, an isotype control. Both antibodies, engineered with cysteine inserted after position 239, were conjugated to linkers D4, D6 and D8 and subsequently reacted with maleimide bearing AZ-1508. The resulting ADCs are shown in Figure 4. Trastuzumab–linker conjugates of D4, D6 and D8 yielded DAR 4, DAR 6 and DAR 8 ADCs and were labeled as T-D4-1508, T-D6-1508 and T-D8-1508, respectively (Figure 4). NIP228–linker conjugates of D4, D6 and D8 yielded DAR4, DAR6 and DAR8 ADCs and were labeled as N-D4-1508, N-D6-1508 and N-D8-1508, respectively (Figure 4).

**Figure 5:**
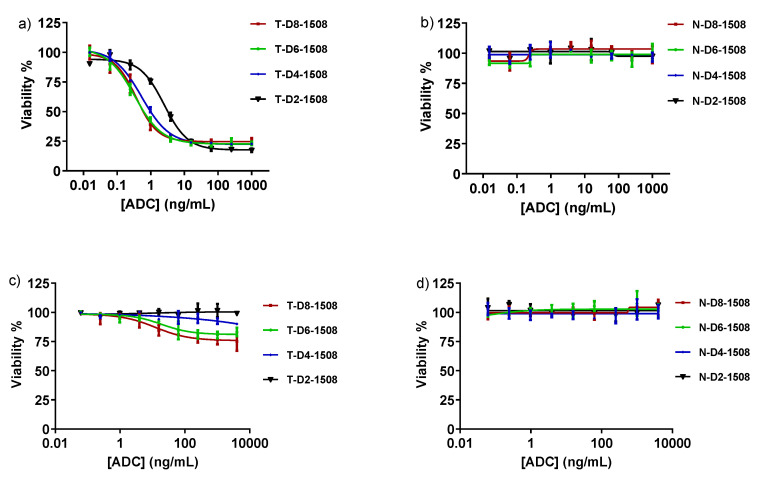
In vitro cytotoxicity assay performed on (**a**) HER2 2+-expressing MDA-MB-361 cell line using site-specifically derived trastuzumab conjugates carrying AZ-1508 (**b**) MDA-MB-361 cell line using site-specifically derived NIP228 conjugates carrying AZ-1508 (**c**) Her2 1+ T47D Cell breast cancer cell line using site-specifically derived trastuzumab conjugates carrying AZ-1508 as the payload (**d**) Her2 1+ T47D Cell breast cancer cell line using site-specifically derived NIP228 conjugates carrying AZ-1508

Post-conjugation, the prepared ADCs were evaluated for their in vitro cytotoxicity. The HER2 2+-expressing MDA-MB-361 and Her2 1+ T47D cell breast cancer cell lines were used for evaluating the ADCs. Figure 5 shows the percentage of viable cells, compared to untreated controls, for cells treated with increasing concentrations of the ADCs for MDA-MB-361 (Figure 5a,b) and T47D cell lines (Figure 5c,d). In these experiments, the cytotoxicity of AZ-1508-derived ADCs with DAR 4, 6 and 8 prepared in a site-specific manner were compared with ADCs (DAR 4, 6 and 8) prepared via classical cysteine conjugation (Supplementary Information) using the same payload (AZ-1508). As expected, the DAR 4 (N-D4-1508), 6 (N-D6-1508) and 8 (N-D8-1508) ADCs derived from NIP228 (isotype control) via a site-specific and classical manner were non-cytotoxic to the MDA-MB-361 and T47D cells at the investigated concentrations (Figure 5b,d). The DAR 4, 6 and 8 ADCs (classical and site-specific) derived from trastuzumab demonstrated significant cytotoxicity to high-HER2-expressing MDA-MB-361 cells. The cytotoxicity of trastuzumab ADCs for site-specific DAR 2 (obtained via maleimide conjugation to cysteine) was found to be lowest followed by DAR 4 (T-D4-1508). The greater toxicity of T-D4-1508 over the DAR 2 counterpart was expected and attributed to the greater drug loading. DAR 6 (T-D6-1508) and DAR 8 (T-D8-1508) conjugates of trastuzumab were found to be equipotent. The equipotency of T-D6-1508 and T-D8-1508 may be explained by the high potency of the AZ-1508 warhead. This trend was also observed for DAR 4, 6 and 8 ADCs derived from trastuzumab via hinge cysteine-based classical conjugation (Supplementary Information). The observed results also indicated that ADCs obtained via the site-specific conjugation method were slightly more cytotoxic than ADCs obtained via the classical method. The cytotoxicity assays using DAR 4, 6 and 8 ADCs (classical and site-specific) performed on the low-HER2-expressing cell line T47D are shown in Figure 5c. The results showed increasing cell toxicity with increasing DAR at a given concentration. Despite the high potency of the AZ-1508 warhead, the decrease in cell viability with the increase in DAR was observed in the T47D cell line as opposed to the MDA-MB-361 cell line due to differences in their HER2 expression. A similar trend was also observed for ADCs derived from trastuzumab and NIP228 via hinge cysteine-based classical conjugation (Figures S1 and S2, Supplementary Information). In vitro cytotoxicity assays were also done on T47D breast cancer cell line for trastuzumab and NIP228 via hinge cysteine-based classical conjugation and the results can be found in the Supplementary Information (Figures S1 and S2). The observed cytotoxicity results suggest that the linker-based two-step approach can lead to well-defined high-DAR ADCs that have equipotency (if not higher) as compared to classical methods.

## 3. Materials and Methods

### 3.1. Synthesis

All reagents were purchased through VWR or Sigma Aldrich (USA) and were used without further purification. ^1^H and ^13^C NMR spectra were obtained on a Bruker Ascend 400 spectrometer. Coupling constants are quoted in hertz (Hz). Mass Spectrometry was obtained using a Waters Acquity UPLC LCMS.

Compound **3.** To a solution of 1 (detailed procedure for synthesis of compound 1 is provided in the Supplementary Information, 0.18 g, 0.37 mmol) in DMF (2 mL), N,N-Diisopropylethylamine (0.3 mL, 1.6 mmol) was added followed by addition of 4-nitrophenyl carbonate derivative of Spiro[2.4]hepta-4,6-dien-1-ol (0.23 g, 0.8 mmol). The reaction mixture was stirred for 4 h at room temperature. DMF was removed under reduced pressure. The residue was purified by silica gel chromatography to give the desired product (0.2 g, 69%) as a white solid. ^1^H NMR (400 MHz, Methanol-*d4*) δ 7.77–7.69 (br d, 2H), 7.62–7.52 (br d, 2H), 7.35–7.27 (br d, 2H), 7.27–7.19 (m, 2H), 6.42–6.34 (m, 2H), 6.33–6.27 (m, 2H), 6.21–6.12 (m, 2H), 6.01–5.93 (m, 2H), 4.31–4.09 (m, 5H), 4.07–3.99 (m, 2H), 3.99–3.86 (m, 5H), 3.04 (br s, 8H) 2.35–2.18 (m, 2H), 1.73–1.50 (m, 4H). ^13^C NMR (101 MHz, CD_3_OD) δ 16.1, 25.7, 40.3, 40.5, 41.9, 50.1, 63.2, 66.0, 66.4, 120.0, 125.2, 127.2, 127.8, 128.4, 130.5, 134.9, 139.2, 141.3, 144.2, 156.7, 156.9, 157.2. MS (ESI) m/z calculated for C_42_H_47_N_5_O_10_ [M]^+^ 781.3, found: 782.6 [M+H]^+^. To the resultant Fmoc-protected solution (0.2 g, 0.25 mmol) in DMF (2 mL), diethyl amine (1 mL) was added. The reaction mixture was stirred for 4 h at room temperature. Solvents were removed under reduced pressure. The residue was purified by reverse-phase chromatography to give the desired product (0.12 g, 54%) as a white solid. ^1^H NMR (400 MHz, Methanol-*d4*) δ 6.44–6.35 (m, 2H), 6.33–6.26 (m, 2H), 6.21–6.11 (m, 2H), 6.02–5.90 (m, 2H), 4.31–4.07 (m, 7H), 4.04–3.91 (m, 2H), 3.09 (br s, 8H) 2.35–2.19 (m, 2H), 1.76–1.48 (m, 4H). ^13^C NMR (101 MHz, CD_3_OD) δ 16.2, 25.7, 40.3, 40.9, 41.9, 50.4, 62.1, 66.5, 66.4, 128.6, 130.8, 134.8, 139.2, 156.8, 158.1. MS (ESI) m/z calculated for C_27_H_37_N_5_O_8_ [M]^+^ 559.3, found: 560.6 [M+H]^+^.

Compound **D4.** To a solution of compound 3 (0.12 g, 0.22 mmol), DMF (1 mL) was added and cooled to 0 °C. To the cooled solution, Iodoacetic anhydride dissolved in DMF (1 mL) was added in a dropwise fashion (0.09 g, 0.26 mmol). The resultant solution was allowed to stir for 5 min followed by addition of DIPEA (90 µL, 3.4 mmol). The reaction was stirred at room temperature for an additional 3 h. The residue was subjected to reverse-phase chromatography to yield D4 (0.067 g, 22%) as a white powder. ^1^H NMR (400 MHz, Dimethyl Sulfoxide-*d6*) δ 6.46–6.37 (m, 2H), 6.36–6.29 (m, 2H), 6.29–6.21 (m, 2H), 6.10–6.01 (m, 2H), 4.25–4.11 (m, 2H), 4.05–3.78 (m, 7H), 3.33 (br s, 2H), 2.94 (br s, 8H) 2.35–2.25 (m, 2H), 1.77–1.55 (m, 4H). ^13^C NMR (101 MHz, DMSO-d6) δ 15.8, 25.1, 39.6, 39.7, 41.4, 47.8, 61.8, 64.9, 127.8, 129.8, 134.6, 138.7, 155.4, 155.7, 167.4. MS (ESI) m/z calculated for C_29_H_38_IN_5_O_9_ [M]^+^ 727.2, found: 728.7 [M+H]^+^.

Compound **4.** To a solution of 2 (detailed procedure for synthesis of compound 2 is provided in the Supplementary Information, 1 g, 1.7 mmol) in DMF (5 mL), N,N-Diisopropylethylamine (1.1 mL, 6.1 mmol) was added followed by addition of 4-nitrophenyl carbonate derivative of Spiro[2.4]hepta-4,6-dien-1-ol (1.8 g, 6.1 mmol). The reaction mixture was stirred for 12 h at room temperature. DMF was removed under reduced pressure. The residue was purified by silica gel chromatography to give the desired product (1.1 g, 64%) as a white solid. ^1^H NMR (400 MHz, Methanol-*d4*) δ 7.68–7.55 (br d, 2H), 7.54–7.41 (br d, 2H), 7.26–7.17 (m, 2H), 7.17–7.09 (m, 2H), 6.40–6.29 (m, 3H), 6.30–6.20 (m, 3H), 6.14–6.02 (m, 3H), 5.94–5.81 (m, 3H), 4.42–4.21 (m, 6H), 4.21–4.05 (m, 6H) 3.95–3.82 (m, 3H), 3.05 (br s, 12H), 2.27–2.11 (m, 3H), 1.65–1.40 (m, 6H). ^13^C NMR (101 MHz, CD_3_OD) δ 17.7, 27.1, 43.3, 48.6, 59.4, 64.1, 67.8, 78.8, 121.3, 122.4, 126.6, 128.5, 129.2, 130.0, 132.1, 136.2, 140.5, 142.8, 145.6, 158.6, 159.2. MS (ESI) m/z calculated for C_55_H_63_N_7_O_14_ [M]^+^ 1045.4, found: 1046.6 [M+H]^+^. To a solution of the Fmoc-protected compound (1 g, 1 mmol) in DMF (3 mL), diethyl amine (1 mL) was added. The reaction mixture was stirred for 4 h at room temperature. Solvents were removed under reduced pressure. The residue was purified by reverse-phase chromatography to give the desired product (0.65 g, 79%) as a white solid. ^1^H NMR (400 MHz, Methanol-*d4*) δ 6.64–6.35 (m, 3H), 6.33–6.24 (m, 3H), 6.21–6.12 (m, 3H), 6.0–5.89 (m, 3H), 4.31–4.11 (m, 3H), 4.11–4.01 (m, 6H) 3.01–3.91 (m, 3H), 3.08 (br s, 12H), 2.35–2.21 (m, 3H), 1.73–1.52 (m, 6H). ^13^C NMR (101 MHz, CD_3_OD) δ 15.9, 25.4, 40.0, 40.5, 41.7, 56.2, 63.8, 66.2, 128.3, 130.4, 134.5, 138.9, 156.6, 157.6. MS (ESI) m/z calculated for C_40_H_53_N_7_O_12_ [M]^+^ 823.4, found: 824.1 [M+H]^+^.

Compound **D6.** To a solution of compound 4 (0.1 g, 0.12 mmol), DMF (1 mL) was added and cooled to 0 °C. To the cooled solution, Iodoacetic anhydride dissolved in DMF (1 mL) was added in a dropwise fashion (0.05 g, 0.15 mmol). The resultant solution was allowed to stir for 5 min followed by addition of DIPEA (45 µL, 1.7 mmol). The reaction was stirred at room temperature for an additional 3 h. The residue was subjected to reverse-phase chromatography to yield D6 (0.067 g, 21%) as a white powder. ^1^H NMR (400 MHz, Dimethyl Sulfoxide-*d6*) δ 6.46–6.37 (m, 3H), 6.35–6.29 (m, 3H), 6.28–6.20 (m, 3H), 6.09–5.98 (m, 3H), 4.25–4.02 (m, 9H), 4.03–3.80 (m, 3H) 3.58 (s, 1H), 2.94 (br s, 12H), 2.39–2.22 (m, 3H), 1.78–1.56 (m, 6H). ^13^C NMR (101 MHz, DMSO-d6) δ 16.8, 26.1, 42.4, 58.3, 62.2, 65.9, 128.7, 130.8, 135.6, 139.7, 156.3, 156.4. MS (ESI) m/z calculated for C_42_H_54_IN_7_O_13_ [M]^+^ 991.3, found: 992.5 [M+H]^+^.

Compound **6.** To a solution of **5** (detailed procedure for synthesis of compound **5** is provided in the Supplementary Information, 0.2 g, 0.36 mmol) in DMF (2 mL), N,N-Diisopropylethylamine (0.2 mL, 0.9 mmol) was added followed by addition of 4-nitrophenyl (spiro[2,4]hepta-4,6-dien-1-ylmethyl) carbonate (0.72 g, 0.9 mmol). The reaction mixture was stirred for 12 h at room temperature. DMF was removed under reduced pressure. The residue was purified by silica gel chromatography to give the desired product (0.36 g, 61%) as a white solid. ^1^H NMR (400 MHz, Methanol-*d4*) δ 7.87–7.77 (br d, 2H), 7.72–7.63 (br d, 2H), 7.45–7.37 (m, 2H), 7.37–7.29 (m, 2H), 6.56–6.46 (m, 4H), 6.46–6.36 (m, 4H), 6.32–6.24 (m, 4H), 6.10–6.04 (m, 4H), 4.51–4.27 (m, 8H), 4.26–3.97 (m, 16H) 3.73 (m, 2H), 3.64–3.49 (m, 6H), 3.19 (br s, 16H), 2.55–2.44 (m, 2H), 2.43–2.31 (m, 4H), 1.80–1.60 (m, 8H). ^13^C NMR (101 MHz, CD_3_OD) δ 17.7, 27.2, 42.1, 43.4, 51.6, 64.9, 67.8, 68.0, 71.6, 121.5, 126.7, 128.7, 129.3, 130.0, 132.2, 136.3, 140.7, 142.9, 145.7, 158.8, 159.2. MS (ESI) m/z calculated for C_81_H_102_N_12_O_25_ [M]^+^ 1642.7, found: 1643.3 [M+H]^+^.

Compound **D8.** To a solution of Fmoc-protected 6 (0.36 g, 0.22 mmol) in DMF (2 mL), diethyl amine (1 mL) was added. The reaction mixture was stirred for 4 h at room temperature. Solvents were removed under reduced pressure. The residue was purified by reverse-phase chromatography to give the desired product (0.24 g, 78%) as a white solid. ^1^H NMR (400 MHz, Methanol-*d4*) δ 6.43–6.35 (m, 4H), 6.34–6.24 (m, 4H), 6.22–6.10 (m, 4H), 6.02–5.90 (m, 4H), 4.32–4.13 (m, 6H), 4.10–3.88 (m, 18H) 3.68–3.44 (m, 8H), 3.07 (br s, 16H), 2.46–2.33 (m, 2H), 2.33–2.20 (m, 4H), 1.76–1.51 (m, 8H). ^13^C NMR (101 MHz, CD_3_OD) δ 15.9, 25.4, 40.1, 40.4, 41.7, 49.9, 63.1, 66.1, 66.6, 67.3, 69.9, 128.4, 130.4, 134.5, 138.9, 157.2, 157.8. MS (ESI) m/z calculated for C_66_H_92_N_12_O_23_ [M]^+^ 1420.6, found: 1421.1 [M+H]^+^. The resulting amine solution (0.1 g, 0.07 mmol), DMF (1 mL) was cooled to 0 °C. To this cooled solution, Iodoacetic anhydride dissolved in DMF (1 mL) was added in a dropwise fashion (0.05 g, 0.13 mmol). The resultant solution was allowed to stir for 5 min followed by addition of DIPEA (45 µL, 1.7 mmol). The reaction was stirred at room temperature for an additional 3 h. The residue was subjected to reverse-phase chromatography to yield D8 (0.067 g, 19%) as a white powder. ^1^H NMR (400 MHz, DMSO-*d6*) δ 6.44–6.37 (m, 4H), 6.35–6.30 (m, 4H), 6.29–6.22 (m, 4H), 6.09–6.01 (m, 4H), 4.20–4.02 (m, 6H), 3.99–3.73 (m, 18H) 3.35–3.48 (m, 4H), 3.37–3.25 (m, 3H), 3.23–3.03 (m, 2H), 2.93 (br s, 16H), 2.37–2.23 (m, 4H), 1.77–1.54 (m, 8H). ^13^C NMR (101 MHz, DMSO-d6) δ 15.9, 25.4, 40.1, 40.4, 41.7, 49.9, 63.1, 66.1, 66.6, 67.3, 69.9, 128.4, 130.4, 134.5, 138.9, 157.2, 157.8.MS (ESI) m/z calculated for C_68_H_93_IN_12_O_24_ [M]^+^ 1588.6, found: 1589.8 [M+H]^+^.

### 3.2. General Procedure for Linker Conjugation to the Antibody

Branched linkers D4, D6 and D8 were conjugated to the desired antibody in a two-step fashion. First, antibodies were mildly reduced to generate free thiols by adding 96 µL of 50 mM TCEP solution to 5 mL of 3.6 mg/mL antibody solution in 10 mM PBS, pH 7.4, 1 mM EDTA. The resulting solution was gently mixed at 37 °C for 1 h. The reduced antibody was transferred to a slide-a-lyzer dialysis cassette (10 K MWCO) and dialyzed against PBS, 1 mM EDTA, pH 7.4, 4 °C for 24 h with several buffer changes. The reduced antibody was oxidized to reform internal disulfides by addition of dehydroascorbic acid (50 mM stock in DMSO, 20 eq.) followed by gentle mixing for 4 h at room temperature. The pH of the oxidized antibody solution was then adjusted to range between 8 and 8.5 using borate buffer. The final antibody concentration was adjusted between 2 and 4 mg/mL. To the resulting antibody solution, a solution of branched linkers D4, D6 or D8 to the amount of 8–26 equivalents (10 mM, DMSO) was added. The resulting reaction mixture was briefly vortexed and further incubated at 37 °C for 1–3 h. The conjugation mixture was purified using ceramic hydroxyapatite chromatography (CHT).

### 3.3. General Procedure for Payload Conjugation to the Linker Antibody Construct

The fractions of the linker antibody construct collected by CHT were pooled together and dialyzed into PBS (pH = 6.0). The final antibody concentration was adjusted between 2 and 4 mg/mL. To the resulting solution, a solution of AZ-1508 to the amount of 8–26 equivalents for antibody conjugates of D4, D6 and D8 (10 mM, DMSO) was added. The resulting reaction mixture was briefly vortexed and further incubated at 37 °C for 3 h. The conjugation mixture was purified using ceramic hydroxyapatite chromatography (CHT).

### 3.4. ADC Characterization

Reduced liquid chromatography mass spectrometry analysis (rLCMS), which was used to determine conjugation at the light or heavy chain and the drug to antibody ratio (DAR), was performed on an Agilent 1290 series uHPLC coupled to an Agilent 6230 TOF. An amount of 2 μg of reduced antibodies or ADCs was loaded onto a Zorbax RRHD 300-Diphenyl (2.1 mm × 50 mm, 1.8 μm, Agilent) and eluted at a flow rate of 0.5 mL/min using a step gradient of 80% B after 2.1 min (mobile phase A: 0.1% Formic acid in water, and mobile phase B: 0.1% Formic acid in acetonitrile). A positive time-of-flight MS scan was acquired, and data collection and processing were carried out using MassHunter software (Agilent). Conjugation efficiencies were calculated based on the intensity of the mass spectrometry signals of unconjugated vs. conjugated. For a detailed reduced mass spectrometry analysis of all ADCs and their intermediates, please refer to the Supplementary Information.

### 3.5. In Vitro Cytotoxicity Assays (Materials)

Human breast cancer MDA-MB-361and T47D cell lines were obtained from the American Type Culture Collection (ATCC). The cells were grown in RPMI1640 (Life Technologies, Carlsbad, CA, USA) supplemented with 10% heat-inactivated fetal bovine serum (FBS, Life Technologies) at 37 °C in a humidified 5% CO2 atmosphere. All cell lines were authenticated by short tandem repeat (STR) DNA profiling using real-time PCR analyses (IDEXX Bioresearch Laboratories, ME, USA).

### 3.6. In Vitro Cytotoxicity Assays (Procedure)

Two tumor cell lines, T47D and MDA-MB-361, in the exponential growth phase were seeded in 96-well culture plates at 2000, 1600 and 5000 per well in 80 µl, respectively, allowed to adhere overnight and treated on the following day with 20 µl of serial dilutions of ADCs in duplicate. The treated cells were cultured for another 3 days for MDA-MB-361 cells. Then, the cell viability was determined by the CellTiter-Glo Luminescent Viability Assay (Promega, Madison, WI, USA) according to the manufacturer’s protocol. The IC50 of ADC cytotoxicity was determined by using logistic non-linear regression analysis with Prism software (GraphPad Prism 8). For cytotoxicity assay results of all ADCs using cell lines MDA-MB-361 (Figures S1 and S2) and T47D (Figures S3 and S4) using trastuzumab and NIP228 via hinge cysteine-based classical conjugation, please refer to the Supplementary Information.

## 4. Conclusions

In summary, a flexible two-step method using heterotrifunctional branched linkers has been developed that can lead to DAR 4, 6 and 8 ADCs in a site-specific manner. Though the proof of concept was applied to trastuzumab and Nip228 using AZ-1508, this method could be equally capable of yielding ADCs using other cysteine engineered antibodies and payloads.

## Supplementary Materials

The following are available online at https://www.mdpi.com/1422-0067/21/18/6882/s1. Figure S1: In vitro cytotoxicity of Trastuzumab based ADCs obtained via hinge disulfide conjugation using Her2-expressing MDA-MB-361 breast cancer cell line; Figure S2: In vitro cytotoxicity of NIP228 based ADCs obtained via classical cysteine conjugation using Her2-expressing MDA-MB-361 breast cancer cell line; Figure S3: In vitro cytotoxicity of Trastuzumab based ADCs obtained via hinge disulfide conjugation using Her2-expressing T47D breast cancer cell line; Figure S4: In vitro cytotoxicity of NIP228 based ADCs obtained via classical cysteine conjugation using Her2-expressing T47D breast cancer cell line.

## Abbreviations

ADC	Antibody–Drug Conjugate
DAR	Drug to Antibody Ratio
