# Supplementary material for: Design and Validation of Linkers for Site-Specific Preparation of Antibody–Drug Conjugates Carrying Multiple Drug Copies Per Cysteine Conjugation Site
Source: Int J Mol Sci. 2020 Sep 19;21(18):6882. doi: 10.3390/ijms21186882 (PMC7555909; doi:10.3390/ijms21186882)
Supplement: Supplementary file 1 [file ijms-21-06882-s001.pdf]

Supplemental Information

Design and validation of linkers for site-specific preparation of antibody drug conjugates carrying multiple drug copies per cysteine conjugation site

Amit Kumar [1], Shenlan Mao[2], Nazzareno Dimasi[1] and Changshou Gao[1,*]

[1] Antibody Discovery and Protein Engineering Department, AstraZeneca R&D, Gaithersburg, Maryland 20878, United States

[2] AstraZeneca Oncology R&D, Gaithersburg, Maryland 20878, United States

*Correspondence: changshou.gao@astrazeneca.com

Table of Content

Experimental Design, Materials, and Methods 2

Molecular weight calculations for mass spectrometry analysis 14

General procedure for linker conjugation to the antibody 17

General procedure for payload conjugation to the linker antibody construct 17

ADC characterization 17

In vitro cytotoxicity assays (Materials) 30

In vitro cytotoxicity assays (Procedure) 30

References 32

Experimental Design, Materials, and Methods

General information.

All reagents were purchased through VWR or Sigma Aldrich and were used without further purification. ^{1}H and ^{13}C NMR spectra were obtained on a Bruker Ascend 400 spectrometer. Coupling constants are quoted in hertz (Hz). Mass Spectrometry was obtained using a Waters Acquity UPLC LCMS.

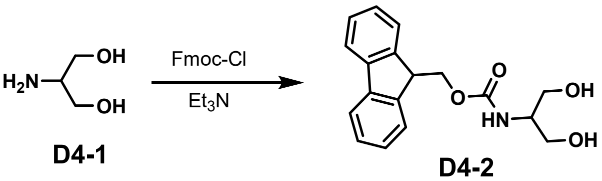

D4-1 **D4-2**

Compound **D4-2** was synthesized as per the published procedure[1]. Briefly, to a solution of serinol (0.91 g, 10 mmol) in DMF (10 mL) was added triethylamine (1.6 mL, 11 mmol) followed by Fluorenylmethyloxycarbonyl chloride (Fmoc-Cl2.5 g, 10 mmol) at 0 °C. The reaction mixture was stirred for 2 h at room temperature. DMF was removed under reduced pressure. The residue was purified by silica gel chromatography (5%-10% methanol in dichloromethane) to give the desired product (2.1 g, 70 %) as a white solid. MS (ESI) m/z calculated for $C_{18}H_{19}NO_4$ $[M]^+$ 313.1, found: 312.2 $[M+H]^+$.

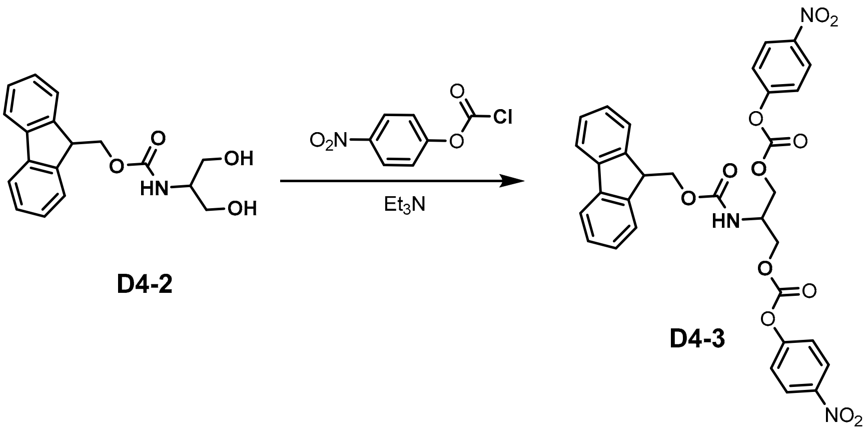

To a solution of Fmoc protected serinol (**D4-2**, 1.0 g, 3.20 mmol) in DMF (5 mL) was added N,N-Diisopropylethylamine (1.2 mL, 6.4 mmol) followed by 4-Nitrophenyl chloroformate (1.4 g, 7.4 mmol) at 0 °C. The reaction mixture was stirred for 4 h at room temperature. DMF was removed under reduced pressure. The residue was purified by silica gel chromatography to give the desired product (1.2 g, 57 %) as a white solid. ^{1}H NMR (400 MHz, Chloroform-*d*) δ 8.20-8.10 (br d, 4H), 7.74-7.65 (br d, 2H), 7.63-7.53 (br d, 2H), 7.32-7.21 (m, 8H), 3.78-3.59 (m, 8H), 6.05 (br s, 1H), 4.59-4.38 (m, 6H), 4.33-4.16 (m, 2H). ^{13}C NMR (101 MHz, $CDCl_3$) δ 47.1, 48.9, 67.2, 120.1, 121.7, 125.0, 125.3, 127.1, 127.8, 141.3, 143.8, 145.4, 152.3, 155.3, 156.2.

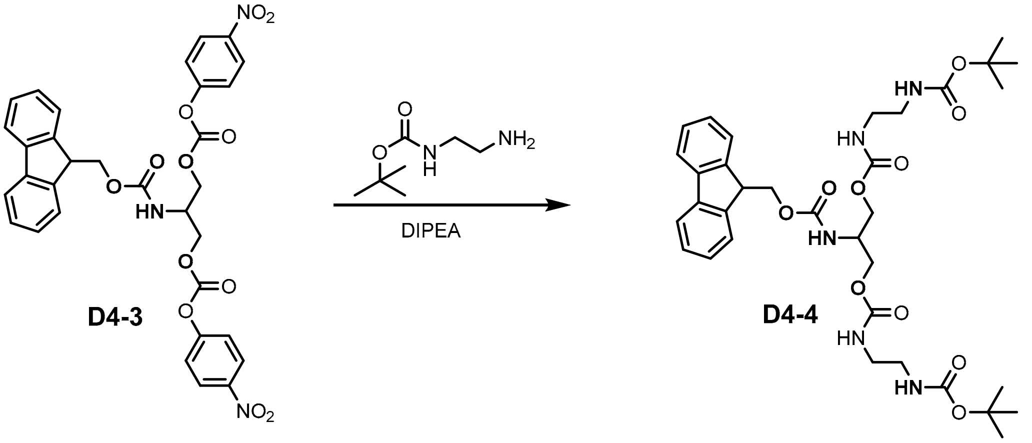

To a solution of **D4-3** (0.5 g, 0.78 mmol) in DMF (2 mL) was added N,N-Diisopropylethylamine (0.3 mL, 1.6 mmol) followed by N-Boc-ethylenediamine (0.26 g, 6.4 mmol) at 0 °C. The reaction mixture was stirred for 6 h at room temperature. DMF was removed under reduced pressure. The residue was purified by silica gel chromatography to give the desired product (0.38 g, 72 %) as a white solid. ^{1}H NMR (400 MHz, Dimethyl Sulfoxide-*d6*) δ 7.83-7.77 (br d, 2H), 7.68-7.54 (m, 2H), 7.41-7.31 (m, 2H), 7.30-7.19 (m, 2H), 7.11 (br s, 2H), 6.71 (br s, 2H), 4.28-4.12 (m, 3H), 4.03-3.71 (m, 5H), 2.93 (br s, 8H), 1.29 (s, 18H). ^{13}C NMR (101 MHz, DMSO-d6) δ 28.8, 40.8, 47.1, 50.3, 63.3, 65.9, 78.2, 120.5, 127.6, 128.2, 141.2, 144.4, 156.0, 156.4. MS (ESI) m/z calculated for $C_{34}H_{47}N_5O_{10}$ $[M]^+$ 685.3, found: 686.2 $[M+H]^+$.

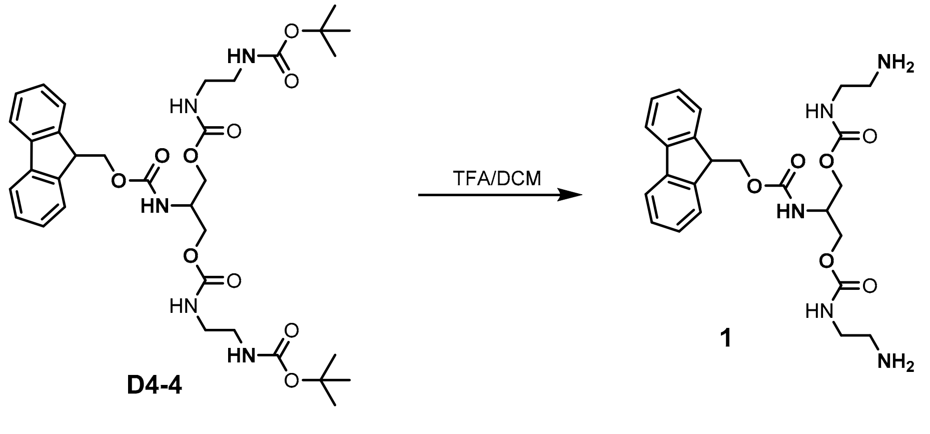

To a solution of **D4-4** (0.3 g, 0.44 mmol) in DCM (2 mL) was added TFA (4 mL) at 0 °C. The reaction mixture was stirred for 4 h at room temperature. Solvents were removed under reduced pressure. The residue was purified by reverse phase chromatography to give the desired product (0.18 g, 85 %) as a white solid. ^{1}H NMR (400 MHz, Methanol-*d4*) δ 7.76-7.67 (br d, 2H), 7.64-7.55 (br d, 2H), 7.43-7.31 (br d, 2H), 7.31-7.24 (m, 2H), 4.43-4.26 (m, 2H), 4.25-3.99 (m, 6H), 3.51-3.34 (m, 2H), 3.17-3.02 (m, 2H). ^{13}C NMR (101 MHz, CD_3OD) δ 41.4, 48.7, 51.4, 51.5, 65.3, 68.4, 121.4, 121.6, 128.6, 129.4, 142.9, 145.6, 158.9, 159.2. MS (ESI) m/z calculated for $C_{24}H_{31}N_5O_6$ $[M]^+$ 485.2, found: 486.1 $[M+H]^+$.

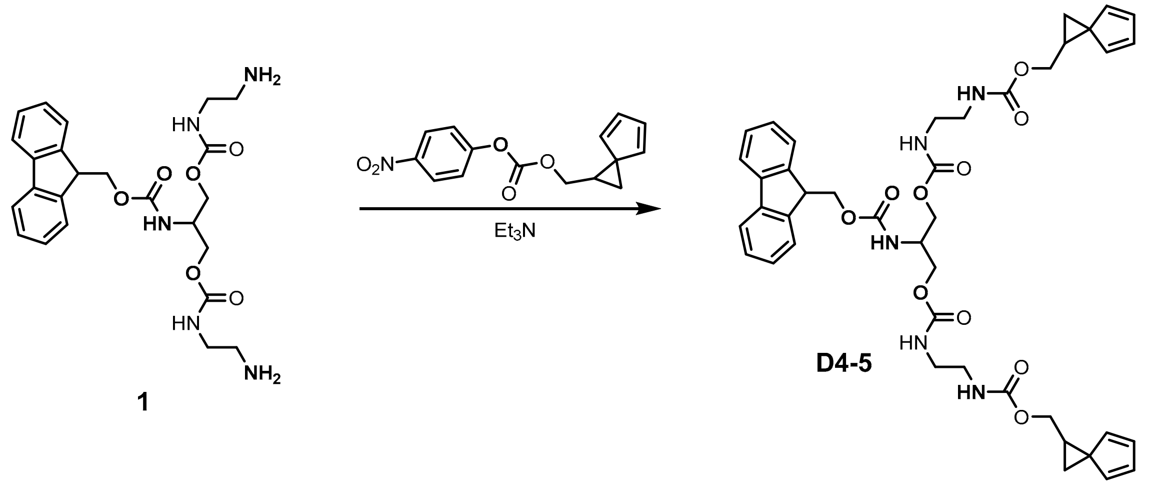

To a solution of **1** (0.18 g, 0.37 mmol) in DMF (2 mL) was added N,N-Diisopropylethylamine (0.3 mL, 1.6 mmol) followed by addition of 4-nitrophenyl carbonate derivative of Spiro[2.4]hepta-4,6-dien-1-ol (0.23 g, 0.8 mmol).[2] The reaction mixture was stirred for 4 h at room temperature. DMF was removed under reduced pressure. The residue was purified by silica gel chromatography to give the desired product (0.2 g, 69 %) as a white solid. ^{1}H NMR (400 MHz, Methanol-*d4*) δ 7.77-7.69 (br d, 2H), 7.62-7.52 (br d, 2H), 7.35-7.27 (br d, 2H), 7.27-7.19 (m, 2H), 6.42-6.34 (m, 2H), 6.33-6.27 (m, 2H), 6.21-6.12 (m, 2H), 6.01-5.93 (m, 2H), 4.31-4.09 (m, 5H), 4.07-3.99 (m, 2H), 3.99-3.86 (m, 5H), 3.04 (br s, 8H) 2.35-2.18 (m, 2H), 1.73-1.50 (m, 4H). ^{13}C NMR (101 MHz, CD_3OD) δ 16.1, 25.7, 40.3, 40.5, 41.9, 50.1, 63.2, 66.0, 66.4, 120.0, 125.2, 127.2, 127.8, 128.4, 130.5, 134.9, 139.2, 141.3, 144.2, 156.7, 156.9, 157.2. MS (ESI) m/z calculated for $C_{42}H_{47}N_5O_{10}$ $[M]^+$ 781.3, found: 782.6 $[M+H]^+$.

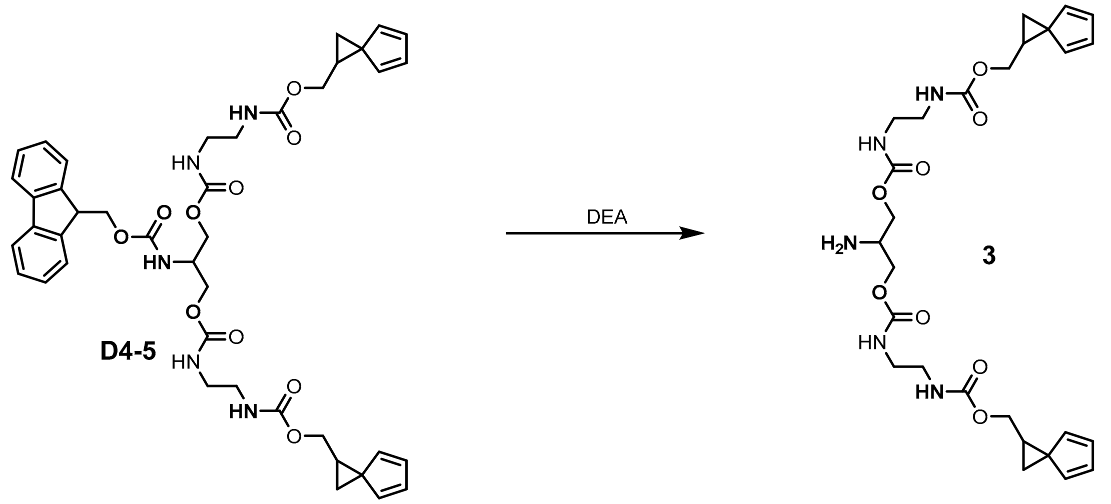

To a solution of Fmoc protected **D4-5** (0.2 g, 0.25 mmol) in DMF (2 mL) was added diethyl amine (1 mL). The reaction mixture was stirred for 4 h at room temperature. Solvents were removed under reduced pressure. The residue was purified by reverse phase chromatography to give the desired product (0.12 g, 54 %) as a white solid. ^{1}H NMR (400 MHz, Methanol-*d4*) δ 6.44-6.35 (m, 2H), 6.33-6.26 (m, 2H), 6.21-6.11 (m, 2H), 6.02-5.90 (m, 2H), 4.31-4.07 (m, 7H), 4.04-3.91 (m, 2H), 3.09 (br s, 8H) 2.35-2.19 (m, 2H), 1.76-1.48 (m, 4H). ^{13}C NMR (101 MHz, CD_3OD) δ 16.2,

25.7, 40.3, 40.9, 41.9, 50.4, 62.1, 66.5, 66.4, 128.6, 130.8, 134.8, 139.2, 156.8, 158.1. MS (ESI) m/z calculated for $C_{27}H_{37}N_5O_8$ $[M]^+$ 559.3, found: 560.6 $[M+H]^+$.

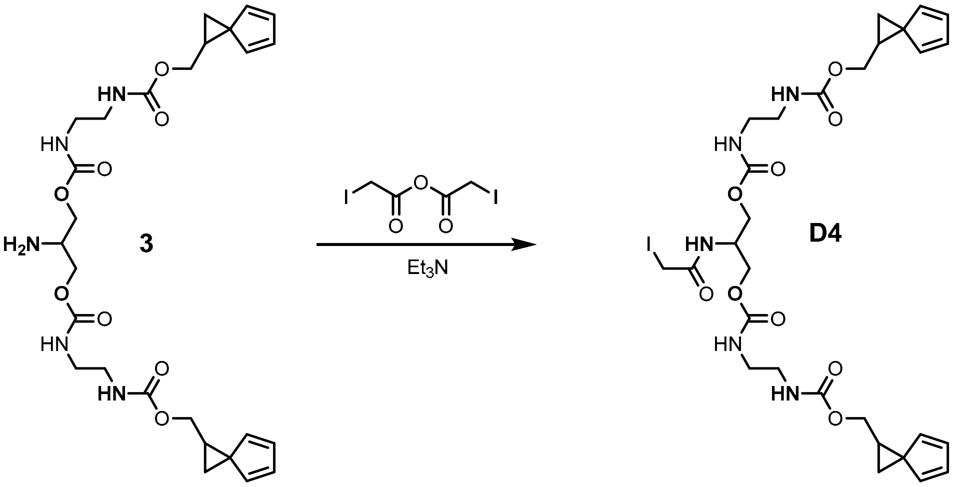

To a solution of compound **3** (0.12 g, 0.22 mmol) was added to DMF (1 mL) and cooled to 0 °C. To the cooled solution Iodoacetic anhydride dissolved in DMF (1 mL) was added in a dropwise fashion (0.09 g, 0.26 mmol). The resultant solution was allowed to stir for 5 min followed by addition of DIPEA (90 µL, 3.4 mmol). The reaction was stirred at room temperature for additional 3 h. The residue was subjected to reverse phase chromatography to yield **D4** (0.067 g, 22 %) as a white powder. ^{1}H NMR (400 MHz, Dimethyl Sulfoxide-*d6*) δ 6.46-6.37 (m, 2H), 6.36-6.29 (m, 2H), 6.29-6.21 (m, 2H), 6.10-6.01 (m, 2H), 4.25-4.11 (m, 2H), 4.05-3.78 (m, 7H), 3.33 (br s, 2H), 2.94 (br s, 8H) 2.35-2.25 (m, 2H), 1.77-1.55 (m, 4H). ^{13}C NMR (101 MHz, DMSO-d6) δ 15.8, 25.1, 39.6, 39.7, 41.4, 47.8, 61.8, 64.9, 127.8, 129.8, 134.6, 138.7, 155.4, 155.7, 167.4. MS (ESI) m/z calculated for $C_{29}H_{38}IN_5O_9$ $[M]^+$ 727.2, found: 728.7 $[M+H]^+$.

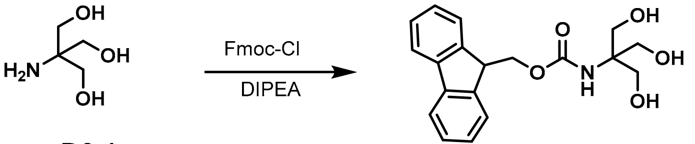

D6-1 D6-2

Compound **D6-1** was synthesized as per the published procedure[3]. Briefly, To a solution of tris (hydroxymethyl)aminomethane (**D6-1**, 5g, 42 mmol) in DMF (20 mL) was added DIPEA (11 mL, 63 mmol) followed by Fluorenylmethyloxycarbonyl chloride (12 g, 46 mmol) at 0 °C. The reaction mixture was stirred for 8 h at room temperature. DMF was removed under reduced pressure. The residue was purified by silica gel chromatography (5%-10% methanol in dichloromethane) to give the desired product (9.8 g, 68 %) as a white solid. MS (ESI) m/z calculated for $C_{19}H_{21}NO_5$ $[M]^+$ 343.1, found: 344.2 $[M+H]^+$.

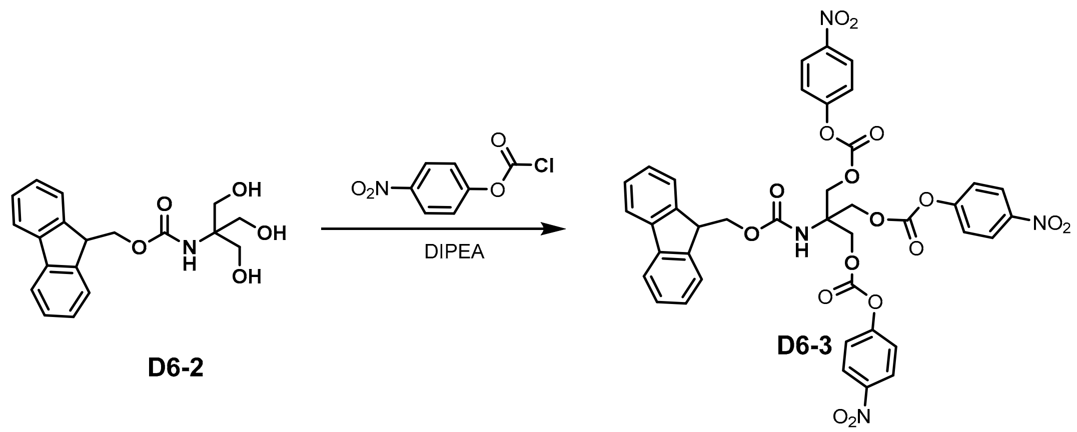

To a solution of Fmoc protected tris (hydroxymethyl)aminomethane (**D6-2**, 4 g, 11.7 mmol) in DMF (40 mL) was added N,N-Diisopropylethylamine (7.2 mL, 38.6 mmol) followed by 4-Nitrophenyl chloroformate (7.8 g, 38.6 mmol) at 0 °C. The reaction mixture was stirred for 8 h at room temperature. DMF was removed under reduced pressure. The residue was purified by silica gel chromatography to give the desired product (5.6 g, 57 %). ^{1}H NMR (400 MHz, Chloroform-*d*) δ 8.22-8.13 (br d, 6H), 7.75-7.68 (br d, 2H), 7.64-7.56 (br d, 2H), 7.39-7.21 (m, 10H), 4.93-4.65 (m, 6H), 4.60-4.37 (m, 2H), 4.28-4.18 (m, 1H). ^{13}C NMR (101 MHz, $CDCl_3$) δ 47.0, 57.1, 60.6, 66.4, 120.1, 121.7, 125.3, 127.1, 127.9, 141.3, 143.7, 145.5, 152.1, 155.2.

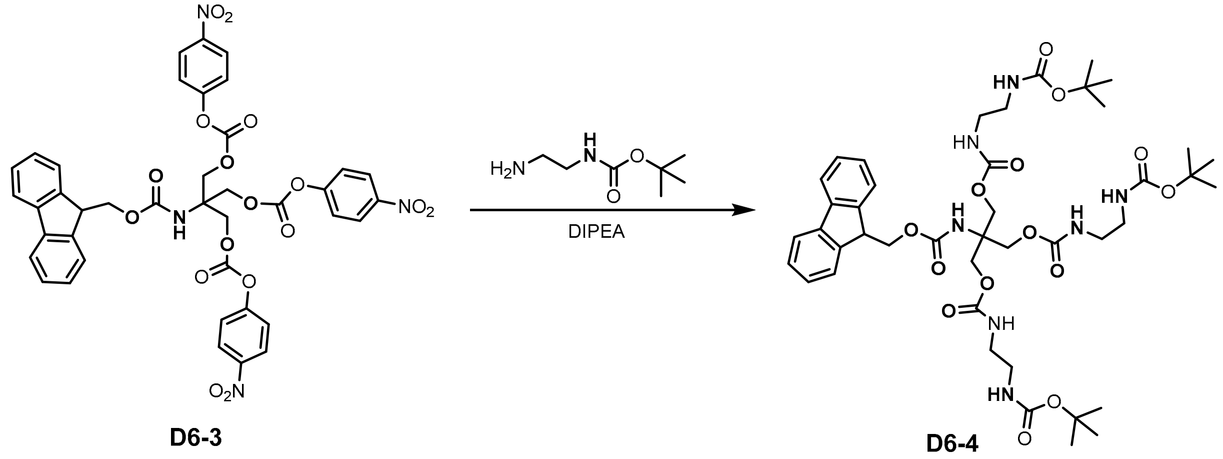

To a solution of **D6-3** (4 g, 4.8 mmol) in DMF (20 mL) was added N,N-Diisopropylethylamine (3.2 mL, 17.2 mmol) followed by N-Boc-ethylenediamine (2.8 g, 6.4 mmol) at 0 °C. The reaction mixture was stirred for 12 h at room temperature. DMF was removed under reduced pressure. The residue was purified by silica gel chromatography to give the desired product (3.2 g, 61 %) as a white solid. ^{1}H NMR (400 MHz, Methanol-*d4*) δ 7.75-7.67 (br d, 2H), 7.64-7.56 (br d, 2H), 7.38-7.24 (m, 4H), 4.83-4.71 (m, 2H), 4.50-4.20 (m, 6H), 4.20-4.11 (m, 1H), 3.18 (m, 12H), 4.20-1.41

(m, 27H). ^{13}C NMR (101 MHz, CD_3OD) δ 27.6, 39.9, 47.0, 57.9, 62.4, 66.4, 78.8, 119.7, 120.7, 125.0, 126.8, 127.5, 141.2, 143.9, 157.0. MS (ESI) m/z calculated for $C_{43}H_{63}N_7O_{14}$ $[M]^+$ 901.4, found: 902.8 $[M+H]^+$.

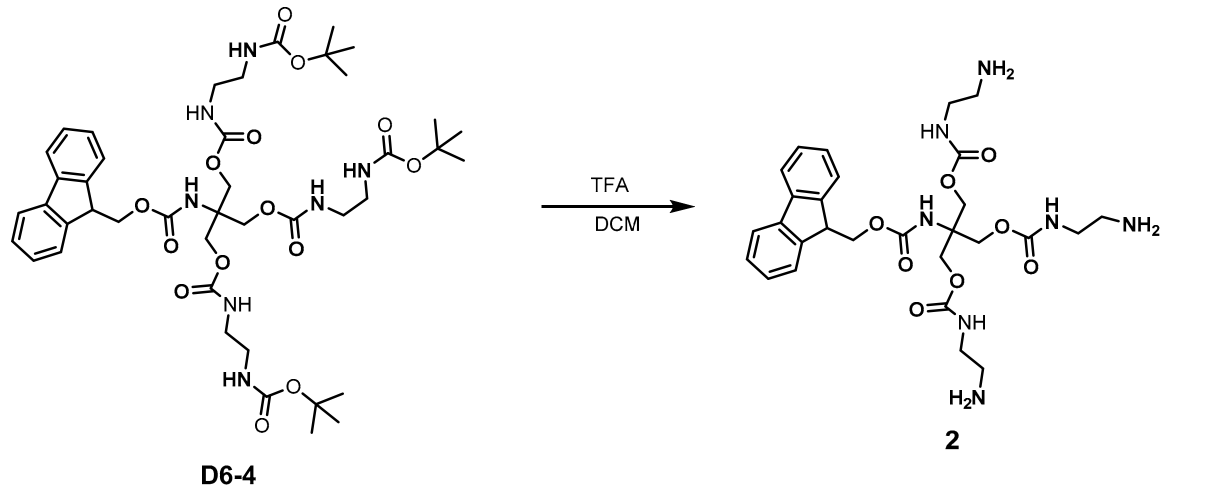

To a solution of **D6-4** (2 g, 2.2 mmol) in DCM (3 mL) was added TFA (6 mL) at 0 °C. The reaction mixture was stirred for 4 h at room temperature. Solvents were removed under reduced pressure. The residue was purified by reverse phase chromatography to give the desired product (1.1 g, 83 %). ^1H NMR (400 MHz, Methanol-*d4*) δ 7.70-7.62 (br d, 2H), 7.57-7.48 (br d, 2H), 7.32-7.23 (m, 2H), 7.23-7.15 (m, 2H), 4.39-4.13 (m, 8H), 4.11-4.03 (m, 1H), 3.36-3.24 (m, 6H), 3.05-2.90 (m, 6H). MS (ESI) m/z calculated for $C_{28}H_{39}N_7O_8$ $[M]^+$ 601.3, found: 602.61 $[M+H]^+$.

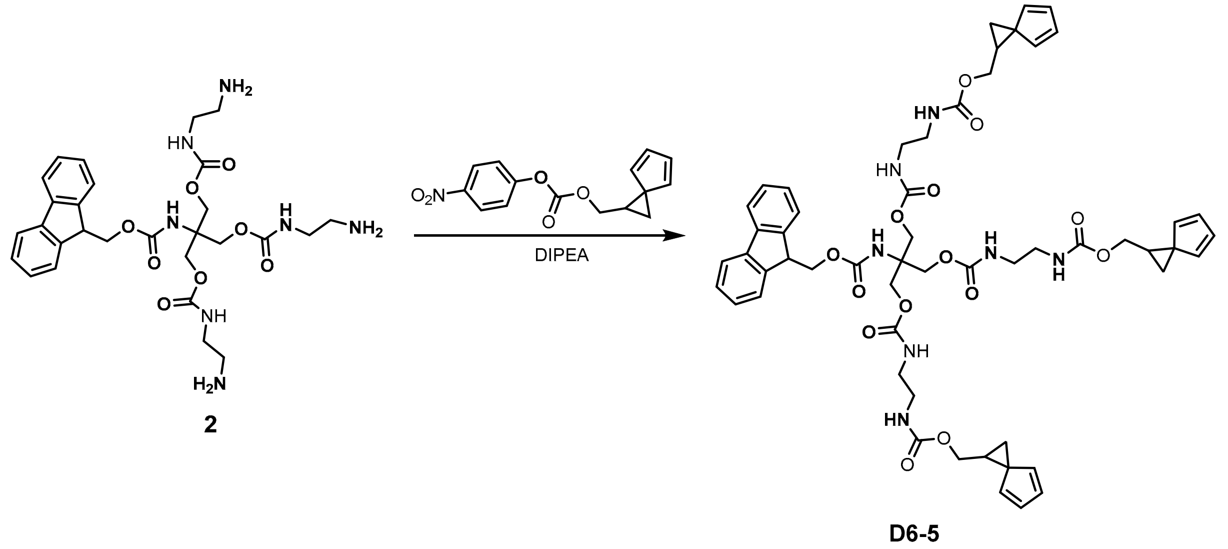

To a solution of **2** (1 g, 1.7 mmol) in DMF (5 mL) was added N,N-Diisopropylethylamine (1.1 mL, 6.1 mmol) followed by addition of 4-nitrophenyl carbonate derivative of Spiro[2.4]hepta-4,6-dien-1-ol (1.8 g, 6.1 mmol). The reaction mixture was stirred for 12 h at room temperature. DMF was removed under reduced pressure. The residue was purified by silica gel chromatography to give the desired product (1.1 g, 64 %) as a white solid. ^1H NMR (400 MHz, Methanol-*d4*) δ 7.68-

7.55 (br d, 2H), 7.54-7.41 (br d, 2H), 7.26-7.17 (m, 2H), 7.17-7.09 (m, 2H), 6.40-6.29 (m, 3H), 6.30-6.20 (m, 3H), 6.14-6.02 (m, 3H), 5.94-5.81 (m, 3H), 4.42-4.21 (m, 6H), 4.21-4.05 (m, 6H) 3.95-3.82 (m, 3H), 3.05 (br s, 12H), 2.27-2.11 (m, 3H), 1.65-1.40 (m, 6H). ^{13}C NMR (101 MHz, CD_3OD) δ 17.7, 27.1, 43.3, 48.6, 59.4, 64.1, 67.8, 78.8, 121.3, 122.4, 126.6, 128.5, 129.2, 130.0, 132.1,136.2, 140.5, 142.8, 145.6, 158.6, 159.2. MS (ESI) m/z calculated for $C_{55}H_{63}N_7O_{14}$ $[M]^+$ 1045.4, found: 1046.6 $[M+H]^+$.

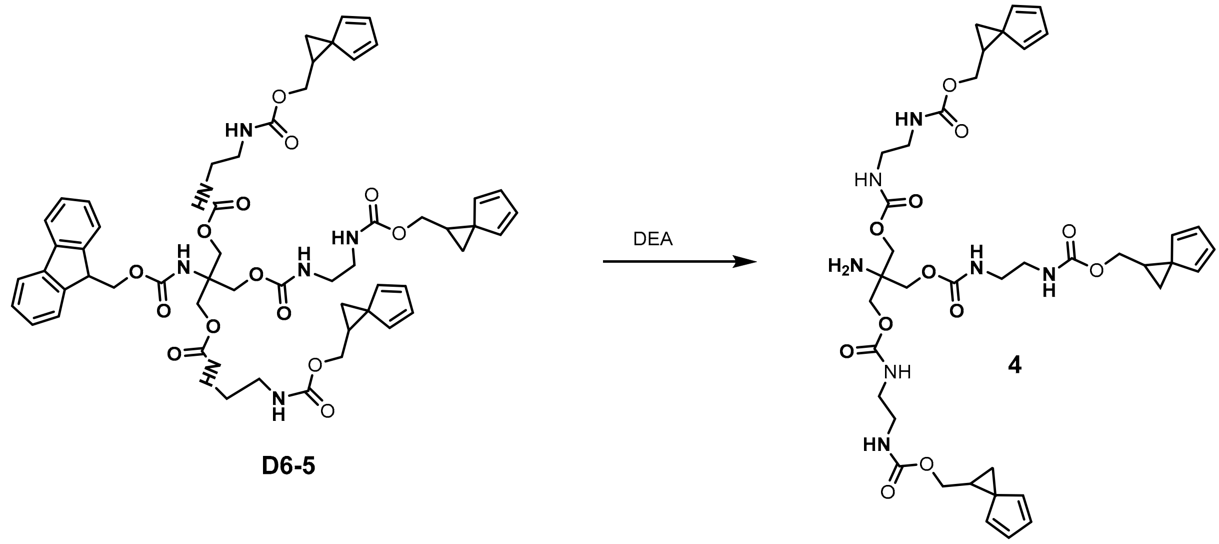

To a solution of Fmoc protected **D4-5** (1 g, 1 mmol) in DMF (3 mL) was added diethyl amine (1 mL). The reaction mixture was stirred for 4 h at room temperature. Solvents were removed under reduced pressure. The residue was purified by reverse phase chromatography to give the desired product (0.65 g, 79 %) as a white solid. ^{1}H NMR (400 MHz, Methanol-*d4*) δ 6.64-6.35 (m, 3H), 6.33-6.24 (m, 3H), 6.21-6.12 (m, 3H), 6.0-5.89 (m, 3H), 4.31-4.11 (m, 3H), 4.11-4.01 (m, 6H) 3.01-3.91 (m, 3H), 3.08 (br s, 12H), 2.35-2.21 (m, 3H), 1.73-1.52 (m, 6H). ^{13}C NMR (101 MHz, CD_3OD) δ 15.9, 25.4, 40.0, 40.5, 41.7, 56.2, 63.8, 66.2, 128.3, 130.4, 134.5, 138.9, 156.6, 157.6. MS (ESI) m/z calculated for $C_{40}H_{53}N_7O_{12}$ $[M]^+$ 823.4, found: 824.1 $[M+H]^+$.

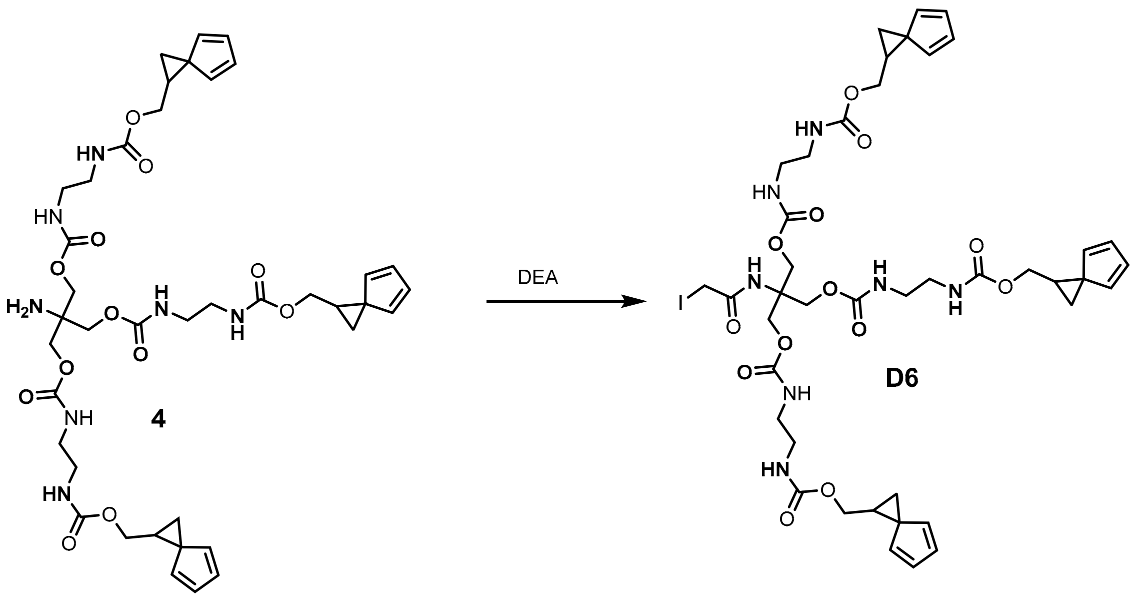

To a solution of compound **4** (0.1 g, 0.12 mmol) was added to DMF (1 mL) and cooled to 0 °C. To the cooled solution Iodoacetic anhydride dissolved in DMF (1 mL) was added in a dropwise fashion (0.05 g, 0.15 mmol). The resultant solution was allowed to stir for 5 min followed by addition of DIPEA (45 µL, 1.7 mmol). The reaction was stirred at room temperature for additional 3 h. The residue was subjected to reverse phase chromatography to yield **D6** (0.067 g, 21 %) as a white powder. ^{1}H NMR (400 MHz, Dimethyl Sulfoxide-*d6*) δ 6.46-6.37 (m, 3H), 6.35-6.29 (m, 3H), 6.28-6.20 (m, 3H), 6.09-5.98 (m, 3H), 4.25-4.02 (m, 9H), 4.03-3.80 (m, 3H) 3.58 (s, 1H), 2.94 (br s, 12H), 2.39-2.22 (m, 3H), 1.78-1.56 (m, 6H). ^{13}C NMR (101 MHz, DMSO-d6) δ 16.8, 26.1, 42.4, 58.3, 62.2, 65.9, 128.7, 130.8, 135.6, 139.7, 156.3, 156.4. MS (ESI) m/z calculated for $C_{42}H_{54}IN_{7}O_{13}$ $[M]^+$ 991.3, found: 992.5 $[M+H]^+$.

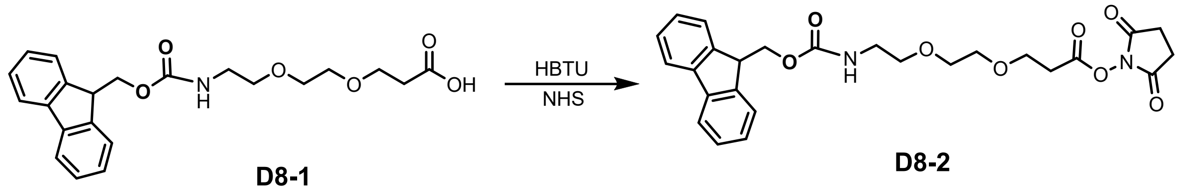

D8-1 **D8-2**

Compound **D8-2** was synthesized as per published procedure.[4] Briefly, To a solution of Fmoc protected **D8-1** (2 g, 5 mmol) in DMF (5 mL) was added N-Hydroxysuccinimide (0.7 g, 6 mmol) followed by addition of HBTU (2.3 g, 6 mmol). The reaction mixture was stirred for 12 h at room temperature. Solvents were removed under reduced pressure. The residue was purified by normal phase chromatography to give the desired product (1.6 g, 64 %) as a white solid. MS (ESI) m/z calculated for $C_{26}H_{28}N_{2}O_{8}$ $[M]^+$ 496.2, found: 497.7 $[M+H]^+$.

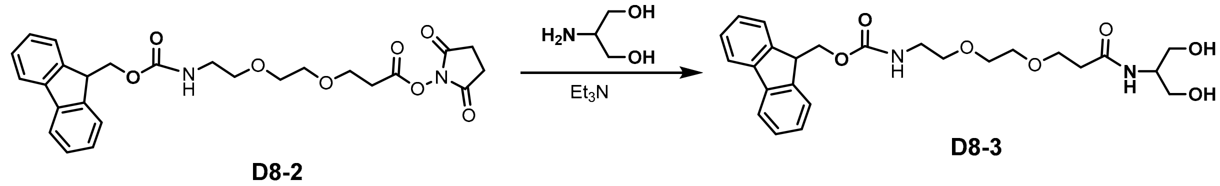

To a solution of NHS activated **D8-2** (1.6 g, 3.2 mmol) in DMF (10 mL) was added triethylamine (0.8 mL, 6 mmol) followed by serinol (0.35 g, 3.8 mmol). The reaction mixture was stirred for 4 h at room temperature. DMF was removed under reduced pressure. The residue was purified by silica gel chromatography (5%-10% methanol in dichloromethane) to give the desired product (1.1 g, 74 %) as a white solid. MS (ESI) m/z calculated for $C_{25}H_{32}N_2O_7$ $[M]^+$ 473.2, found: 474.7 $[M+H]^+$.

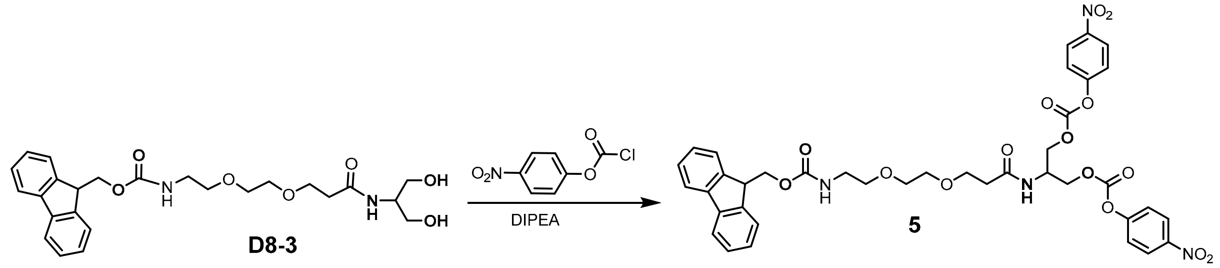

To a solution of Fmoc protected **D8-3** (1.0 g, 2.4 mmol) in DMF (5 mL) was added N,N-Diisopropylethylamine (1.2 mL, 6.4 mmol) followed by 4-Nitrophenyl chloroformate (1.2 g, 5.8 mmol) at 0 °C. The reaction mixture was stirred for 4 h at room temperature. DMF was removed under reduced pressure. The residue was purified by silica gel chromatography to give the desired product (1.3 g, 66 %).

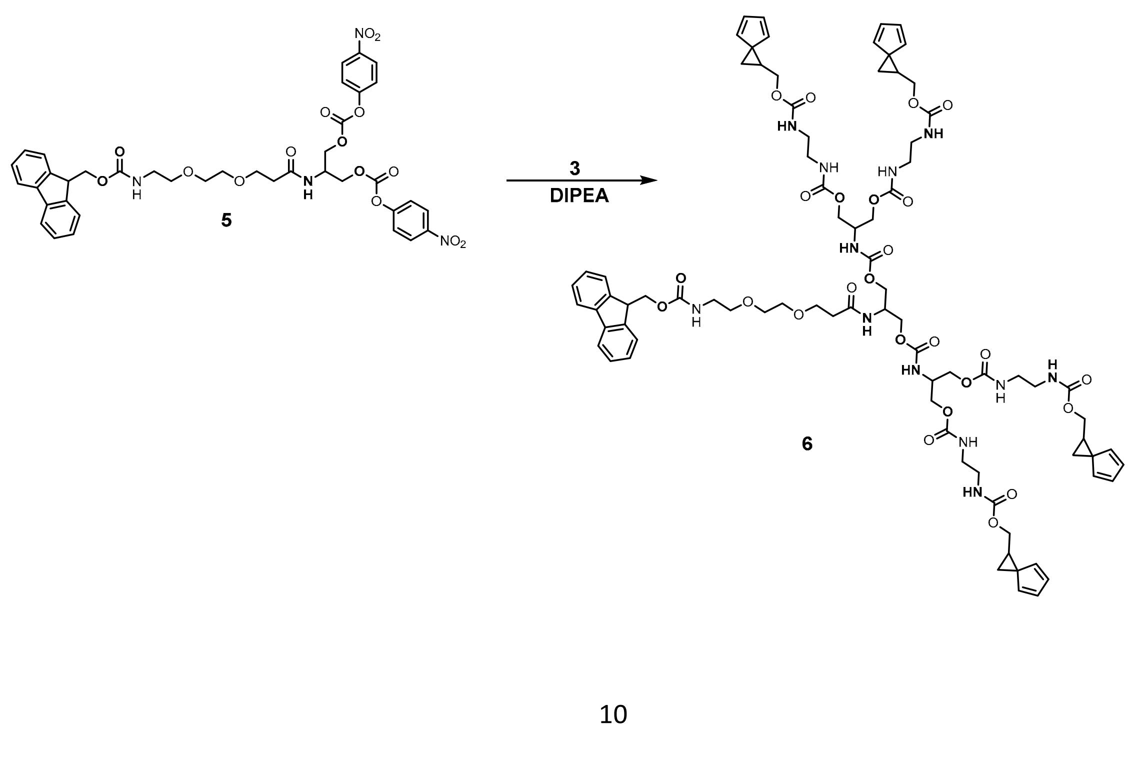

To a solution of **5** (0.2 g, 0.36 mmol) in DMF (2 mL) was added N,N-Diisopropylethylamine (0.2 mL, 0.9 mmol) followed by addition of 4-nitrophenyl carbonate activated **y** (0.72 g, 0.9 mmol). The reaction mixture was stirred for 12 h at room temperature. DMF was removed under reduced pressure. The residue was purified by silica gel chromatography to give the desired product (0.36 g, 61 %) as a white solid. ^{1}H NMR (400 MHz, Methanol-*d4*) δ 7.87-7.77 (br d, 2H), 7.72-7.63 (br d, 2H), 7.45-7.37 (m, 2H), 7.37-7.29 (m, 2H), 6.56-6.46 (m, 4H), 6.46-6.36 (m, 4H), 6.32-6.24 (m, 4H), 6.10-6.04 (m, 4H), 4.51-4.27 (m, 8H), 4.26-3.97 (m, 16H) 3.73 (m, 2H), 3.64-3.49 (m, 6H), 3.19 (br s, 16H), 2.55-2.44 (m, 2H), 2.43-2.31 (m, 4H), 1.80-1.60 (m, 8H). ^{13}C NMR (101 MHz, CD_3OD) δ 17.7, 27.2, 42.1, 43.4, 51.6, 64.9, 67.8, 68.0, 71.6, 121.5, 126.7, 128.7, 129.3, 130.0, 132.2, 136.3, 140.7, 142.9, 145.7, 158.8, 159.2. MS (ESI) m/z calculated for $C_{81}H_{102}N_{12}O_{25}$ $[M]^+$ 1642.7, found: 1643.3 $[M+H]^+$.

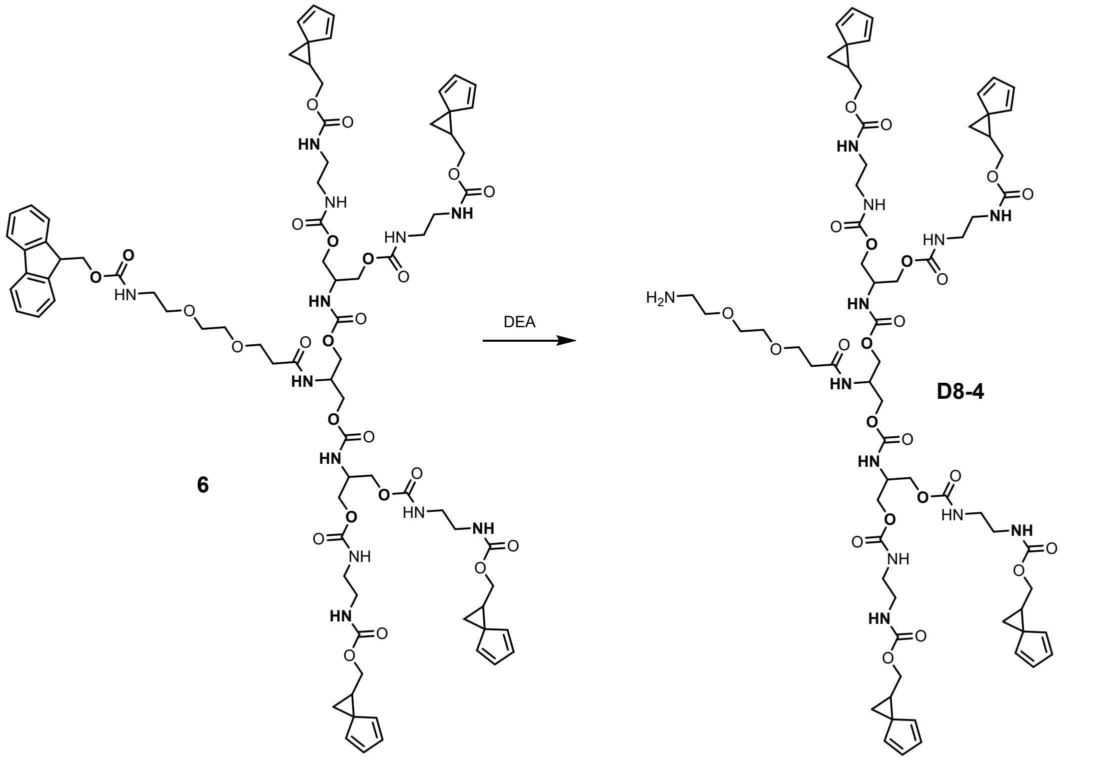

To a solution of Fmoc protected **6** (0.36 g, 0.22 mmol) in DMF (2 mL) was added diethyl amine (1 mL). The reaction mixture was stirred for 4 h at room temperature. Solvents were removed under reduced pressure. The residue was purified by reverse phase chromatography to give the desired product (0.24 g, 78 %) as a white solid. ^{1}H NMR (400 MHz, Methanol-*d4*) δ 6.43-6.35 (m, 4H), 6.34-6.24 (m, 4H), 6.22-6.10 (m, 4H), 6.02-5.90 (m, 4H), 4.32-4.13 (m, 6H), 4.10-3.88 (m, 18H) 3.68-3.44 (m, 8H), 3.07 (br s, 16H), 2.46-2.33 (m, 2H), 2.33-2.20 (m, 4H), 1.76-1.51 (m, 8H). ^{13}C NMR (101 MHz, CD_3OD) δ 15.9, 25.4, 40.1, 40.4, 41.7, 49.9, 63.1, 66.1, 66.6, 67.3, 69.9, 128.4, 130.4, 134.5, 138.9, 157.2, 157.8. MS (ESI) m/z calculated for $C_{66}H_{92}N_{12}O_{23}$ $[M]^+$ 1420.6, found: 1421.1 $[M+H]^+$.

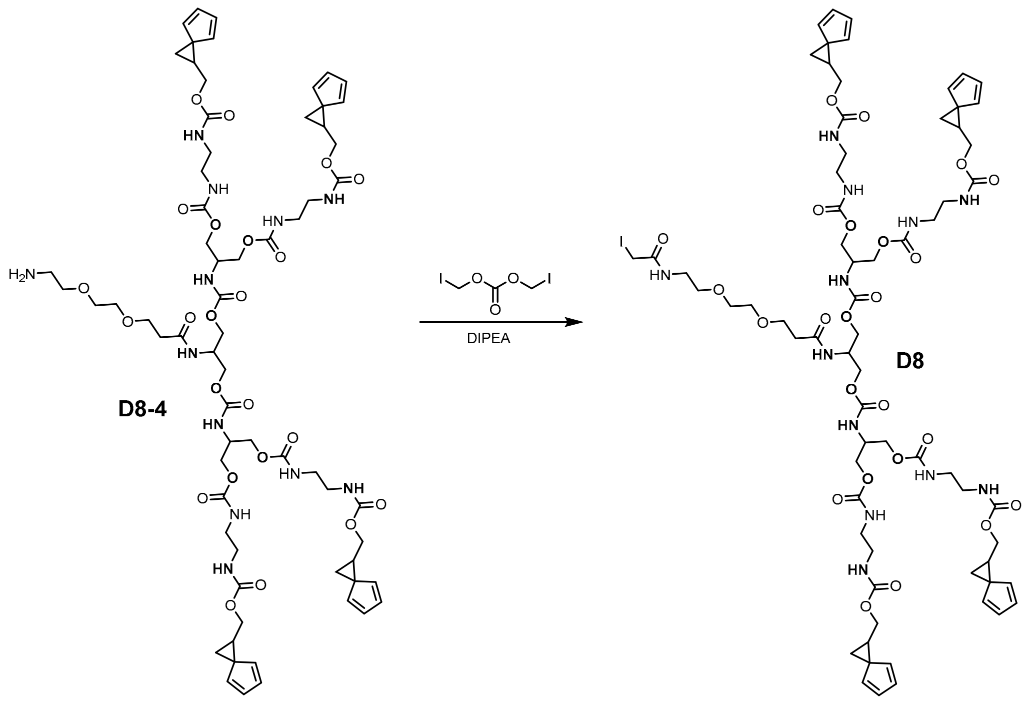

To a solution of compound **5-13** (0.1 g, 0.07 mmol) was added to DMF (1 mL) and cooled to 0 °C. To the cooled solution Iodoacetic anhydride dissolved in DMF (1 mL) was added in a dropwise fashion (0.05 g, 0.13 mmol). The resultant solution was allowed to stir for 5 min followed by addition of DIPEA (45 μL, 1.7 mmol). The reaction was stirred at room temperature for additional 3 h. The residue was subjected to reverse phase chromatography to yield **D8** (0.067 g, 19 %) as a white powder. ^{1}H NMR (400 MHz, DMSO-*d6*) δ 6.44-6.37 (m, 4H), 6.35-6.30 (m, 4H), 6.29-6.22 (m, 4H), 6.09-6.01 (m, 4H), 4.20-4.02 (m, 6H), 3.99-3.73 (m, 18H) 3.35-3.48 (m, 4H), 3.37-3.25 (m, 3H), 3.23-3.03 (m, 2H), 2.93 (br s, 16H), 2.37-2.23 (m, 4H), 1.77-1.54 (m, 8H). ^{13}C NMR (101 MHz, DMSO-d6) δ 15.9, 25.4, 40.1, 40.4, 41.7, 49.9, 63.1, 66.1, 66.6, 67.3, 69.9, 128.4, 130.4, 134.5, 138.9, 157.2, 157.8.MS (ESI) m/z calculated for $C_{68}H_{93}IN_{12}O_{24}$ $[M]^+$ 1588.6, found: 1589.8 $[M+H]^+$.

Molecular weight calculations for mass spectrometry analysis

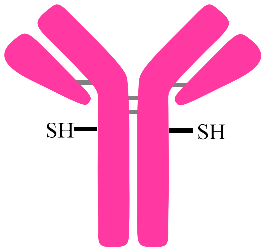

Trastuzumab-S239i (**T**)
Light chain mass: 23433 Da
Heavy chain G0 mass: 50725 Da

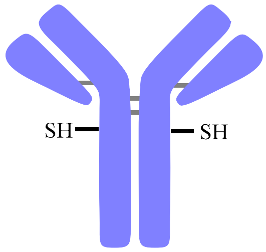

NIP228-S239i (**N**)
Light chain mass: 23191 Da
Heavy chain G0 mass: 50370 Da

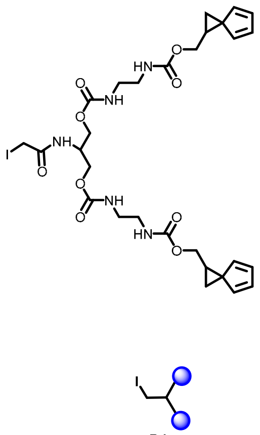

D4

Molecular Weight: 727.55

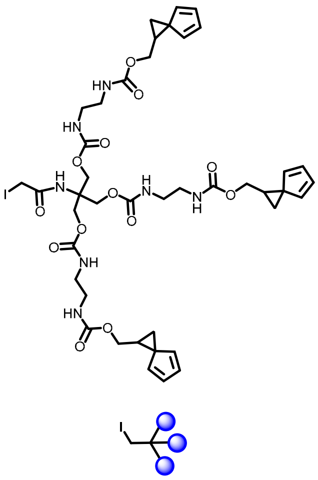

D6

Molecular Weight: 991.83

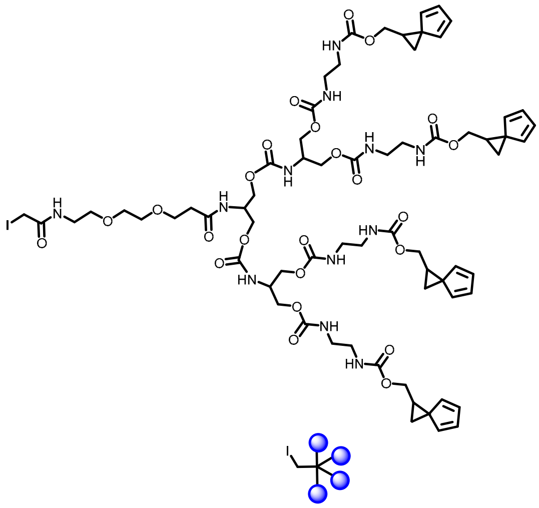

D8

Molecular Weight: 1589.46

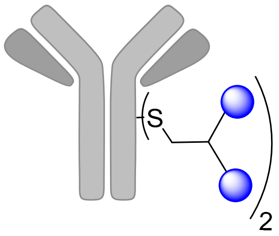

T-D4

Trastuzumab-S239i (**T**)
Light chain mass: 23433 Da
Heavy chain G0 mass: 51325 Da

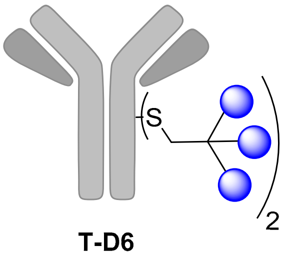

T-D6

Trastuzumab-S239i (**T**)
Light chain mass: 23433 Da
Heavy chain G0 mass: 51589 Da

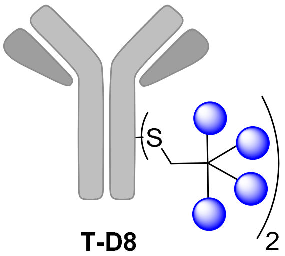

T-D8

Trastuzumab-S239i (**T**)
Light chain mass: 23433 Da
Heavy chain G0 mass: 52187 Da

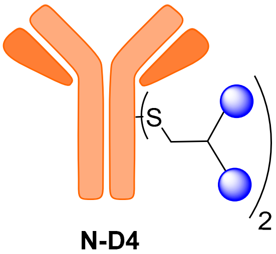

N-D4

NIP228-S239i (**N**)
Light chain mass: 23191 Da
Heavy chain G0 mass: 50970 Da

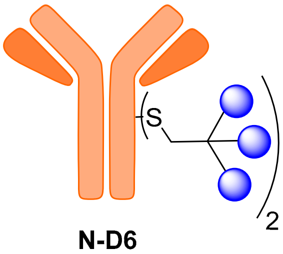

N-D6

NIP228-S239i (**N**)
Light chain mass: 23191 Da
Heavy chain G0 mass: 51234 Da

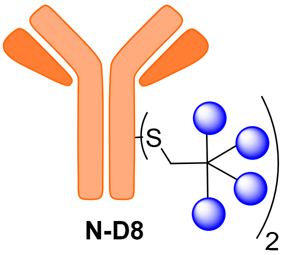

N-D8

NIP228-S239i (**N**)
Light chain mass: 23191 Da
Heavy chain G0 mass: 51832 Da

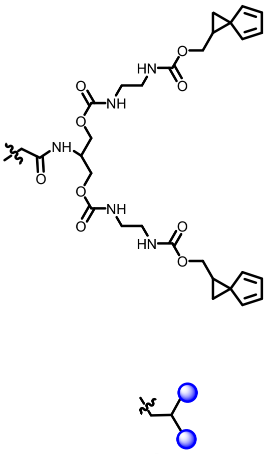

D4

Molecular Weight: 600.65

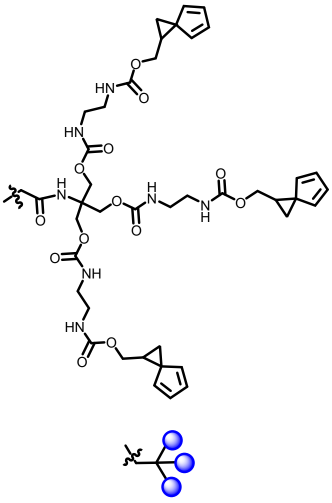

D6

Molecular Weight: 864.38

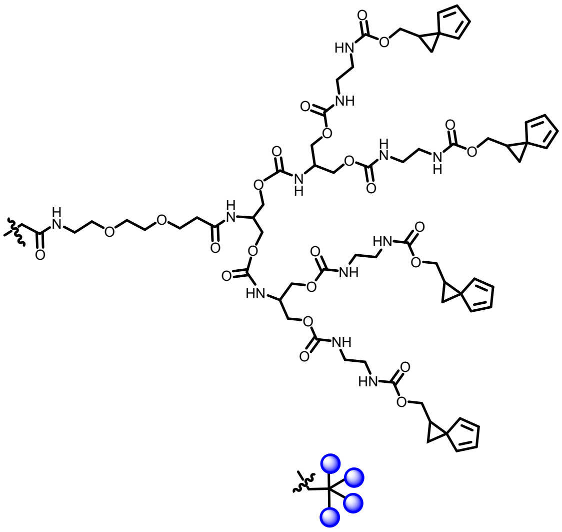

D8

Molecular Weight: 1461.64

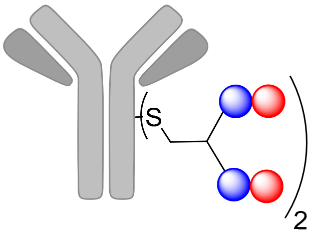

T-D4-1508

Trastuzumab-S239i (**T**)
Light chain mass: 23433 Da
Heavy chain G0 mass: 53509 Da

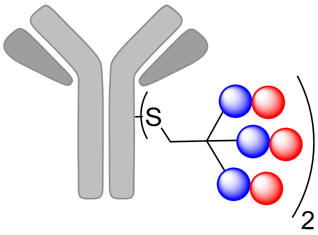

T-D6-1508

Trastuzumab-S239i (**T**)
Light chain mass: 23433 Da
Heavy chain G0 mass: 54865 Da

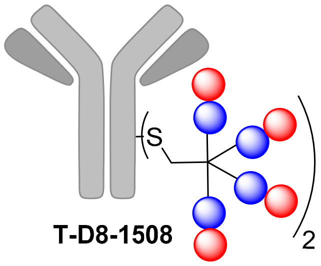

T-D8-1508

Trastuzumab-S239i (**T**)
Light chain mass: 23433 Da
Heavy chain G0 mass: 56555 Da

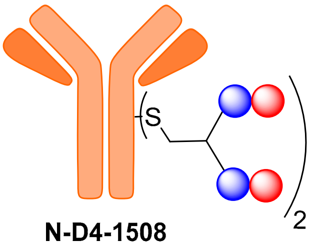

N-D4-1508

NIP228-S239i (**N**)
Light chain mass: 23191 Da
Heavy chain G0 mass: 53154 Da

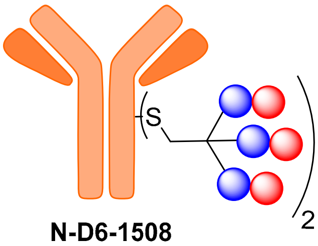

N-D6-1508

NIP228-S239i (**N**)
Light chain mass: 23191 Da
Heavy chain G0 mass: 54510 Da

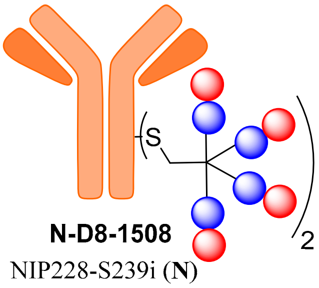

N-D8-1508

NIP228-S239i (**N**)
Light chain mass: 23191 Da
Heavy chain G0 mass: 56200 Da

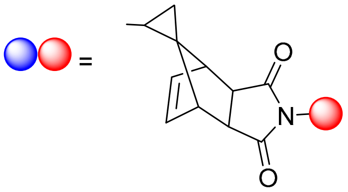

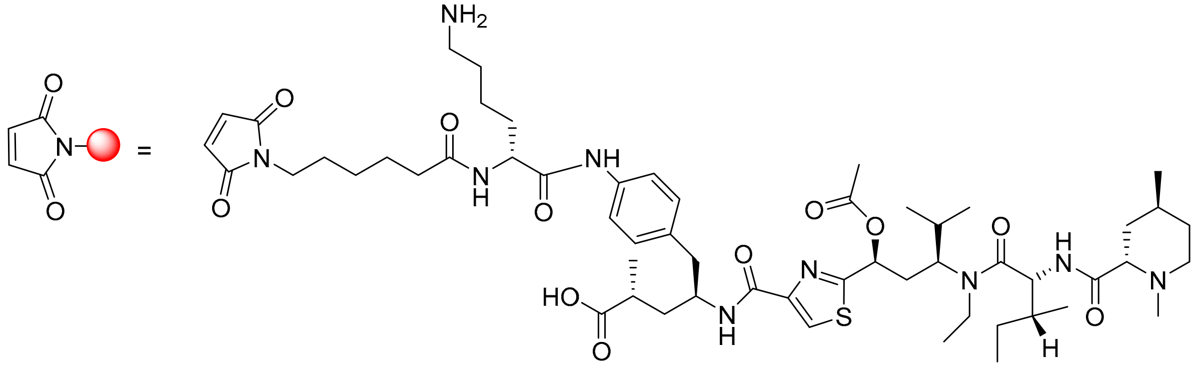

Molecular Weight: 1092.41

General procedure for linker conjugation to the antibody.

Branched linker **D4, D6 and D8** were conjugated to the desired antibody in a two-step fashion. First, antibodies were mildly reduced to generate free thiols by adding 50 mM TCEP solution to 5 mL of 3.6 mg/mL antibody solution in 10 mM PBS, pH 7.4, 1 mM EDTA. The resulting solution was gently mixed at 37 °C for 1 h. Reduced antibody was transferred to a slide-a-lyzer dialysis cassette (10 K MWCO) and dialyzed against PBS, 1 mM EDTA, pH 7.4, 4 °C for 24 h with several buffer changes. Reduced antibody was oxidized to reform internal disulfides by addition of dehydroascorbic acid (50 mM stock in DMSO, 20 eq.) followed by gentle mixing for 4 h at room temperature. The pH of oxidized antibody solution was then adjusted to range between 8 and 8.5 using borate buffer. The final antibody concentration was adjusted between 2 to 4 mg/mL. To the resulting antibody solution was added a solution of branched linker **D4**, **D6** or **D8** to the amount of 8-26 equivalents (10 mM, DMSO). The resulting reaction mixture was briefly vortexed and further incubated at 37 °C for 3 hours. The conjugation mixture was purified using ceramic hydroxyapatite chromatography (CHT).

General procedure for payload conjugation to the linker antibody construct.

The fractions of linker antibody construct collected by CHT were pooled together and dialyzed into PBS (pH = 6.0). The final antibody concentration was adjusted between 2 to 4 mg/mL. To the resulting solution was added a solution of AZ-1508 to the amount of 8-26 equivalents for antibody conjugates of **D4**, **D6** and **D8** (10 mM, DMSO). The resulting reaction mixture was briefly vortexed and further incubated at 37 °C for 3 hours. The conjugation mixture was purified using ceramic hydroxyapatite chromatography (CHT).

ADC characterization

Reduced liquid chromatography mass spectrometry analysis (rLCMS), which was used to determine conjugation at the light or heavy chain and drug to antibody ratio (DAR), was performed on an Agilent 1290 series uHPLC coupled to an Agilent 6230 TOF. 2 µg of reduced antibodies or ADCs were loaded onto a Zorbax RRHD 300-Diphenyl (2.1 × 50 mm, 1.8 µm, Agilent) and eluted at a flow rate of 0.5 mL/min using a step gradient of 80% B after 2.1 min (mobile phase A: 0.1% Formic acid in water and mobile phase B: 0.1% Formic acid in acetonitrile). A positive time-of-flight MS scan was acquired, and data collection and processing were carried out using MassHunter software (Agilent). Conjugation efficiencies were calculated based on intensity of mass spectrometry signals of unconjugated vs conjugated. In the mass spectrum analysis G0, G1 and G2 refer to Fc glycans, the terminal galactose residues present as non-reducing termini.

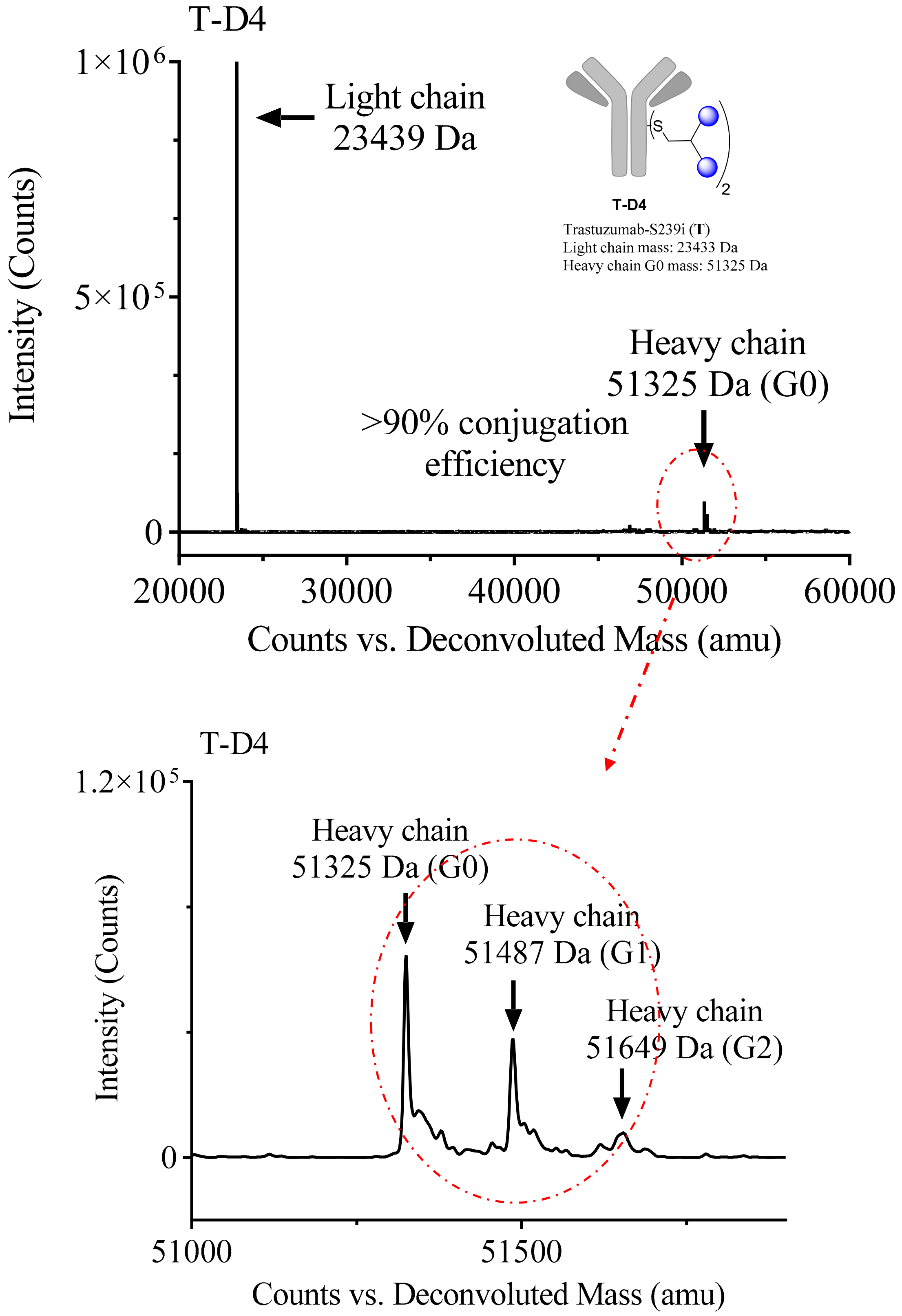
T-D4
1×10^6
5×10^5
0
Intensity (Counts)
Light chain
23439 Da
T-D4
Trastuzumab-S239i (T)
Light chain mass: 23433 Da
Heavy chain G0 mass: 51325 Da
Heavy chain
51325 Da (G0)
>90% conjugation
efficiency
20000
30000
40000
50000
60000
Counts vs. Deconvoluted Mass (amu)
T-D4
1.2×10^5
0
Intensity (Counts)
Heavy chain
51325 Da (G0)
Heavy chain
51487 Da (G1)
Heavy chain
51649 Da (G2)
51000
51500
Counts vs. Deconvoluted Mass (amu)

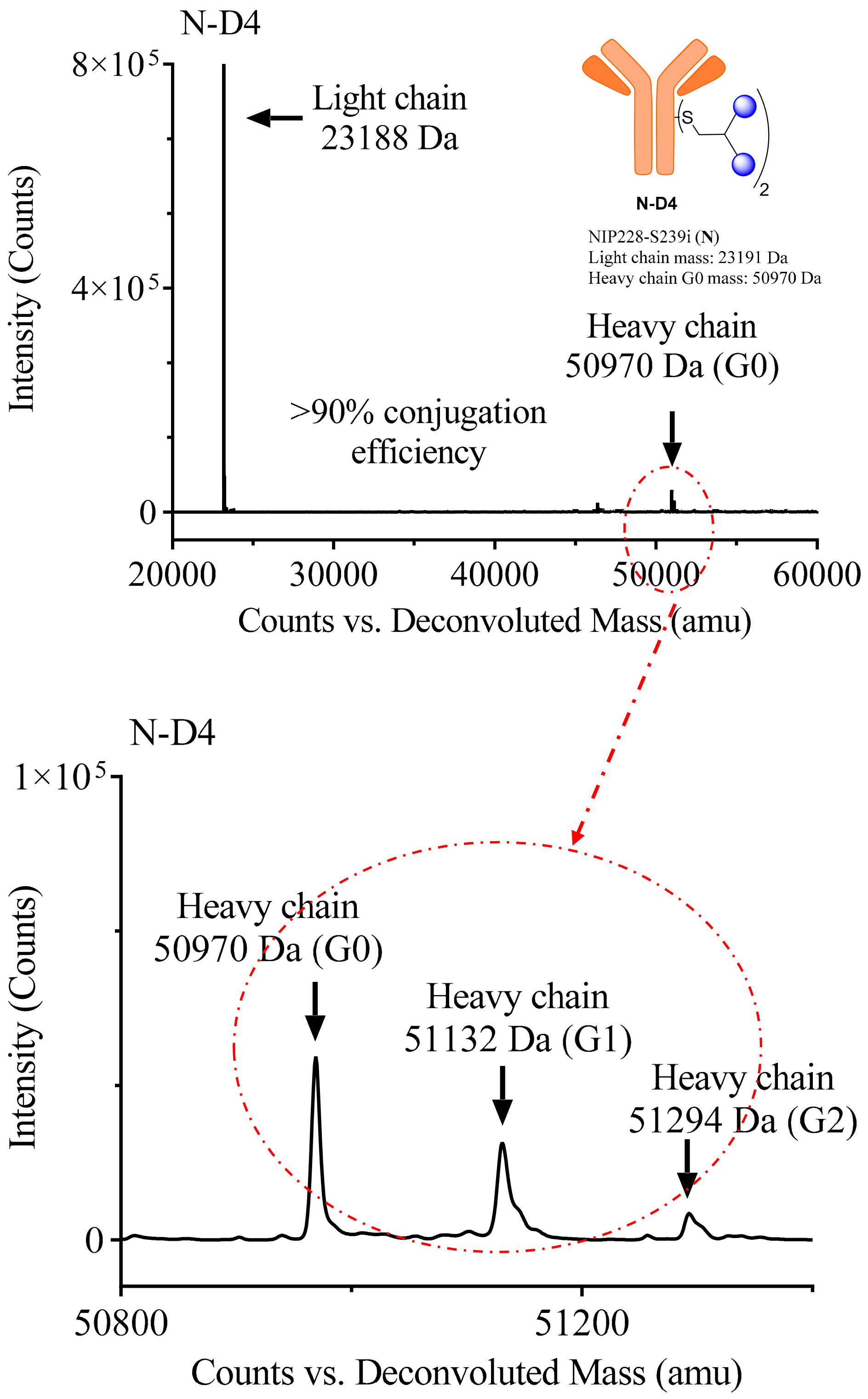
N-D4
8×10^5
4×10^5
0
Light chain
23188 Da
N-D4
NIP228-S239i (N)
Light chain mass: 23191 Da
Heavy chain G0 mass: 50970 Da
Heavy chain
50970 Da (G0)
>90% conjugation
efficiency
20000
30000
40000
50000
60000
Counts vs. Deconvoluted Mass (amu)
Intensity (Counts)
N-D4
1×10^5
0
Heavy chain
50970 Da (G0)
Heavy chain
51132 Da (G1)
Heavy chain
51294 Da (G2)
50800
51200
Counts vs. Deconvoluted Mass (amu)
Intensity (Counts)

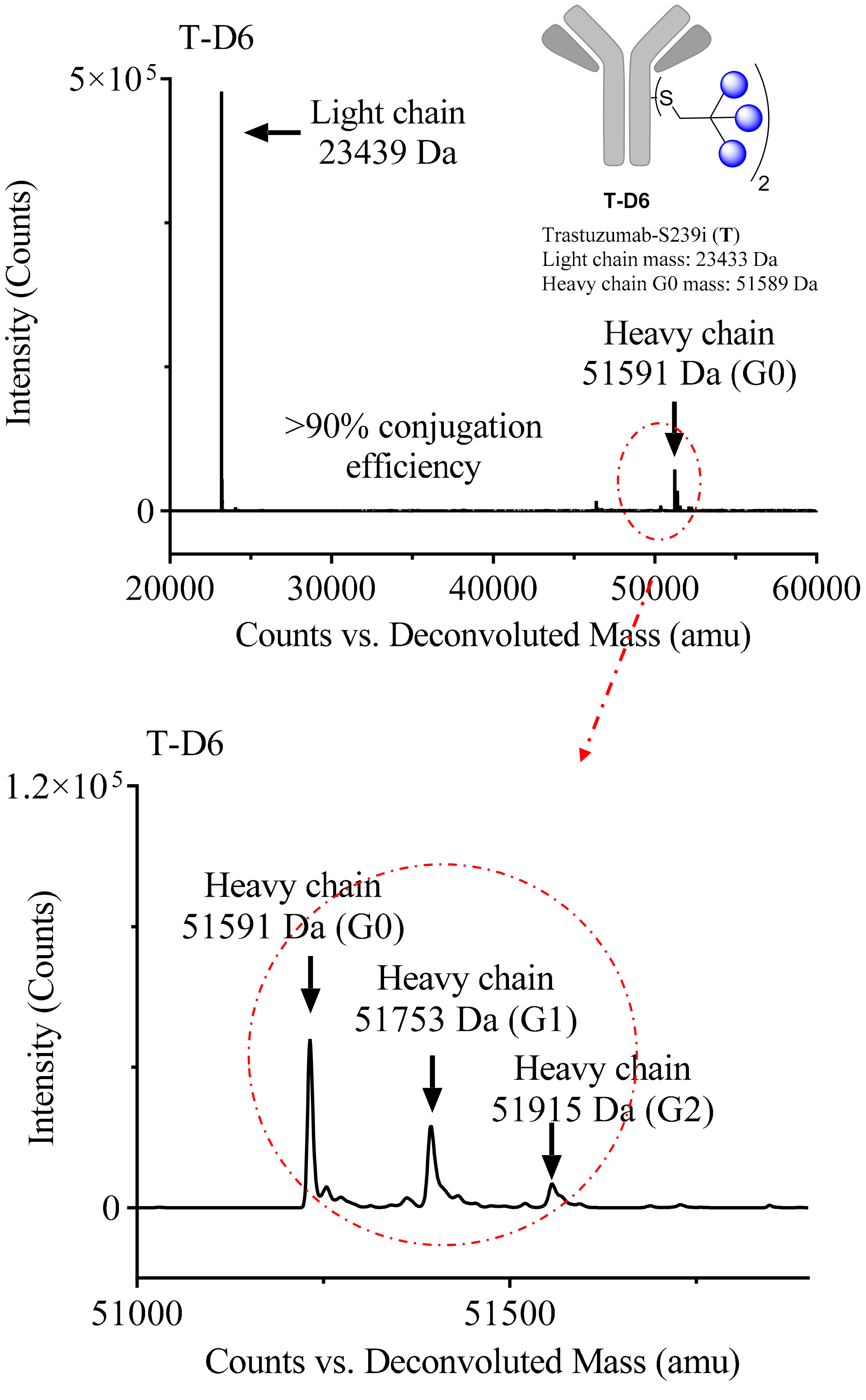
T-D6
5×10^5
0
Light chain
23439 Da
T-D6
Trastuzumab-S239i (T)
Light chain mass: 23433 Da
Heavy chain G0 mass: 51589 Da
Heavy chain
51591 Da (G0)
>90% conjugation
efficiency
Intensity (Counts)
20000
30000
40000
50000
60000
Counts vs. Deconvoluted Mass (amu)
T-D6
1.2×10^5
0
Heavy chain
51591 Da (G0)
Heavy chain
51753 Da (G1)
Heavy chain
51915 Da (G2)
Intensity (Counts)
51000
51500
Counts vs. Deconvoluted Mass (amu)

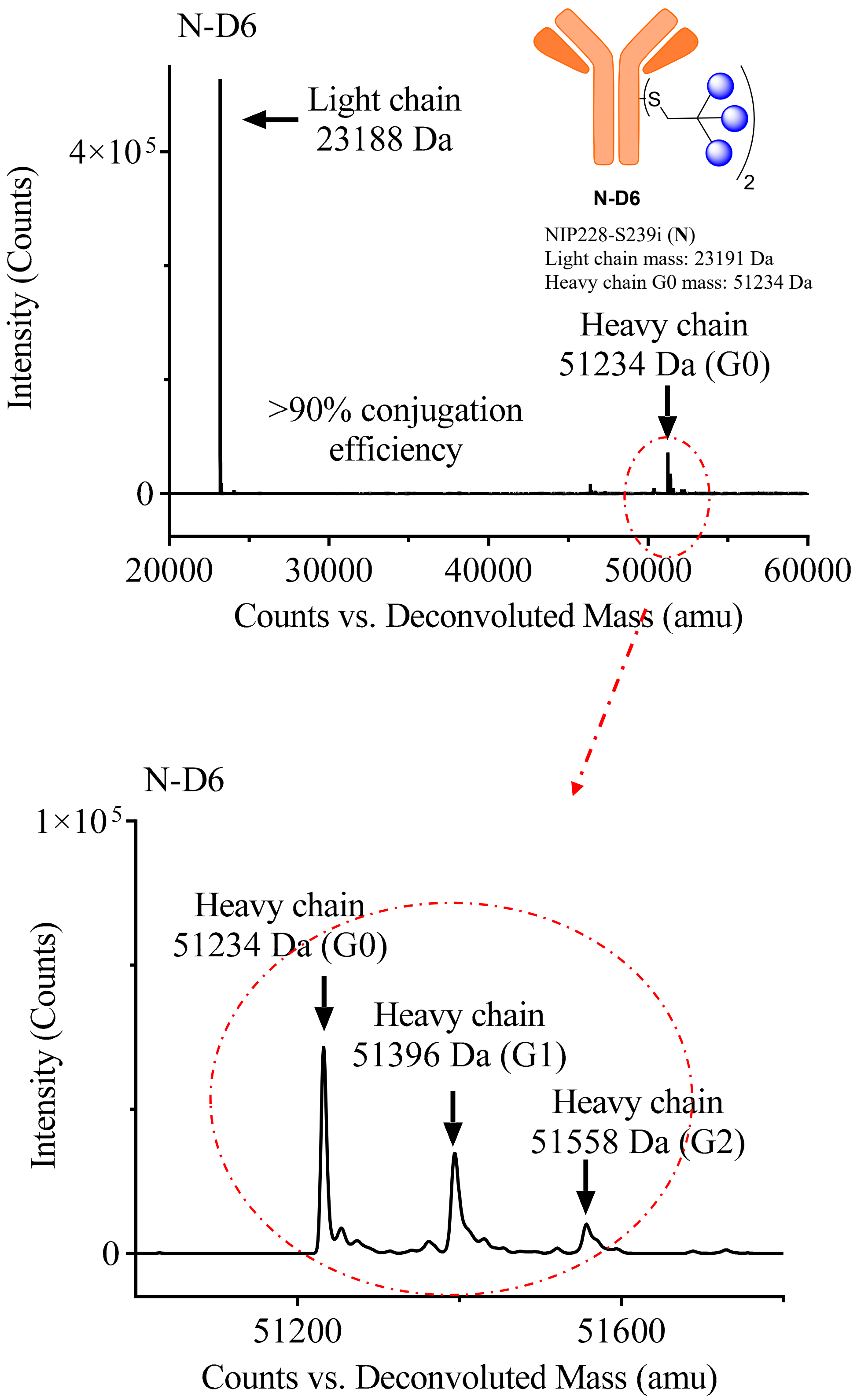
N-D6
Light chain
23188 Da
4×10⁵
0
Intensity (Counts)
N-D6
NIP228-S239i (N)
Light chain mass: 23191 Da
Heavy chain G0 mass: 51234 Da
Heavy chain
51234 Da (G0)
>90% conjugation
efficiency
20000
30000
40000
50000
60000
Counts vs. Deconvoluted Mass (amu)
N-D6
1×10⁵
0
Intensity (Counts)
Heavy chain
51234 Da (G0)
Heavy chain
51396 Da (G1)
Heavy chain
51558 Da (G2)
51200
51600
Counts vs. Deconvoluted Mass (amu)

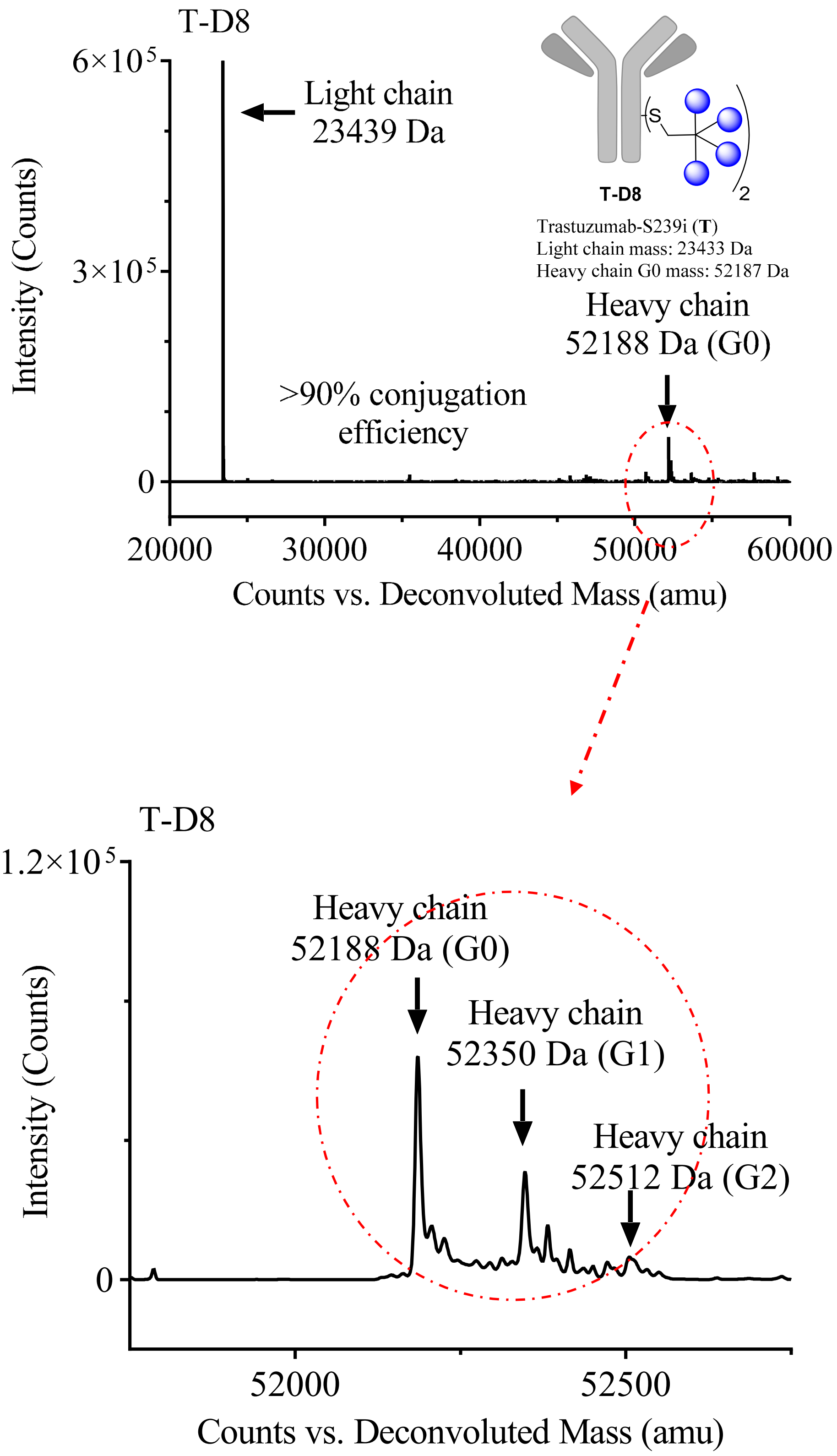
T-D8
6×10^5
3×10^5
0
Light chain
23439 Da
T-D8
2
Trastuzumab-S239i (T)
Light chain mass: 23433 Da
Heavy chain G0 mass: 52187 Da
Heavy chain
52188 Da (G0)
>90% conjugation
efficiency
Intensity (Counts)
20000
30000
40000
50000
60000
Counts vs. Deconvoluted Mass (amu)
T-D8
1.2×10^5
0
Heavy chain
52188 Da (G0)
Heavy chain
52350 Da (G1)
Heavy chain
52512 Da (G2)
Intensity (Counts)
52000
52500
Counts vs. Deconvoluted Mass (amu)

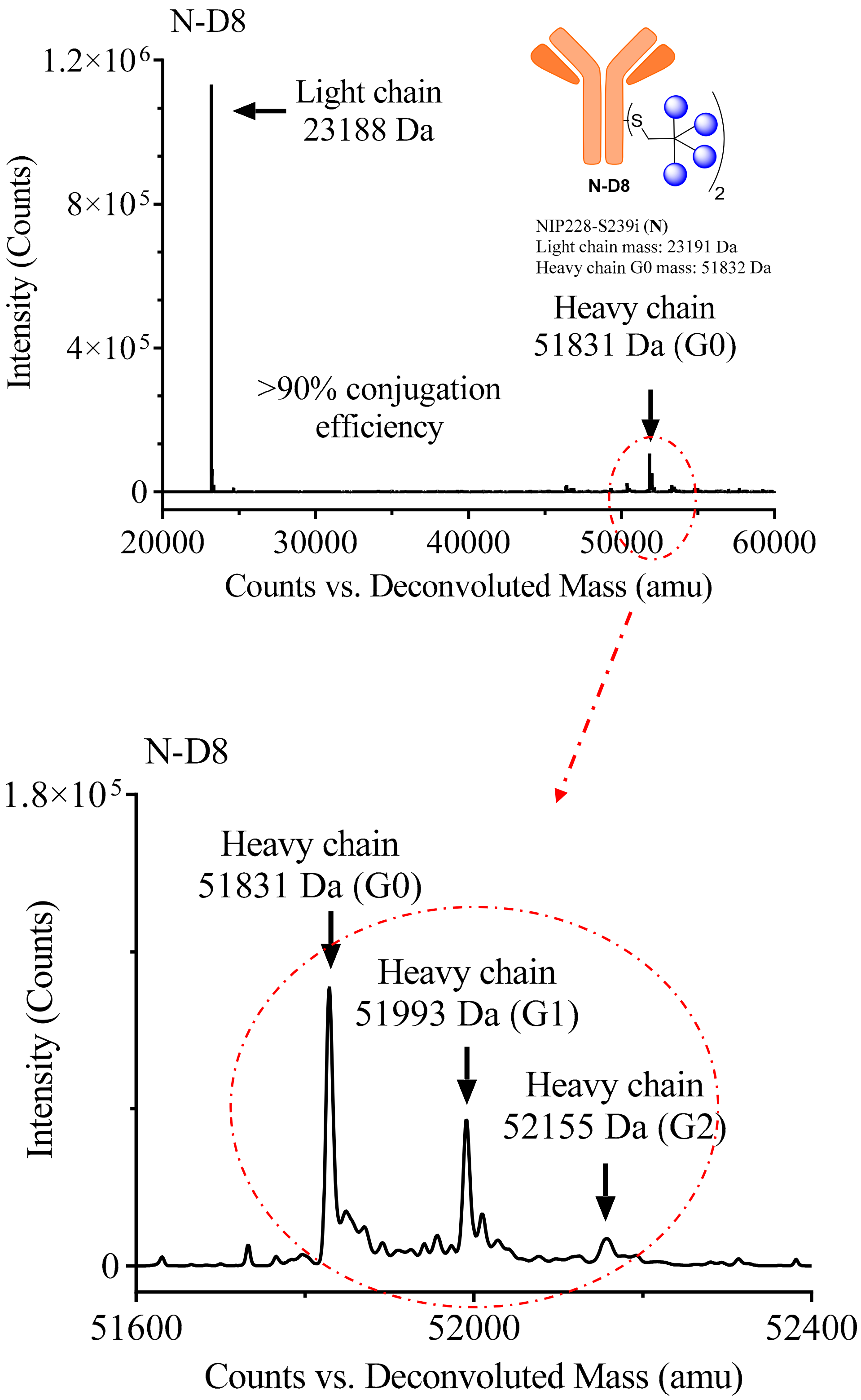
N-D8
1.2×10⁶
8×10⁵
4×10⁵
0
Intensity (Counts)
Light chain
23188 Da
N-D8
NIP228-S239i (N)
Light chain mass: 23191 Da
Heavy chain G0 mass: 51832 Da
Heavy chain
51831 Da (G0)
>90% conjugation
efficiency
20000
30000
40000
50000
60000
Counts vs. Deconvoluted Mass (amu)
N-D8
1.8×10⁵
0
Intensity (Counts)
Heavy chain
51831 Da (G0)
Heavy chain
51993 Da (G1)
Heavy chain
52155 Da (G2)
51600
52000
52400
Counts vs. Deconvoluted Mass (amu)

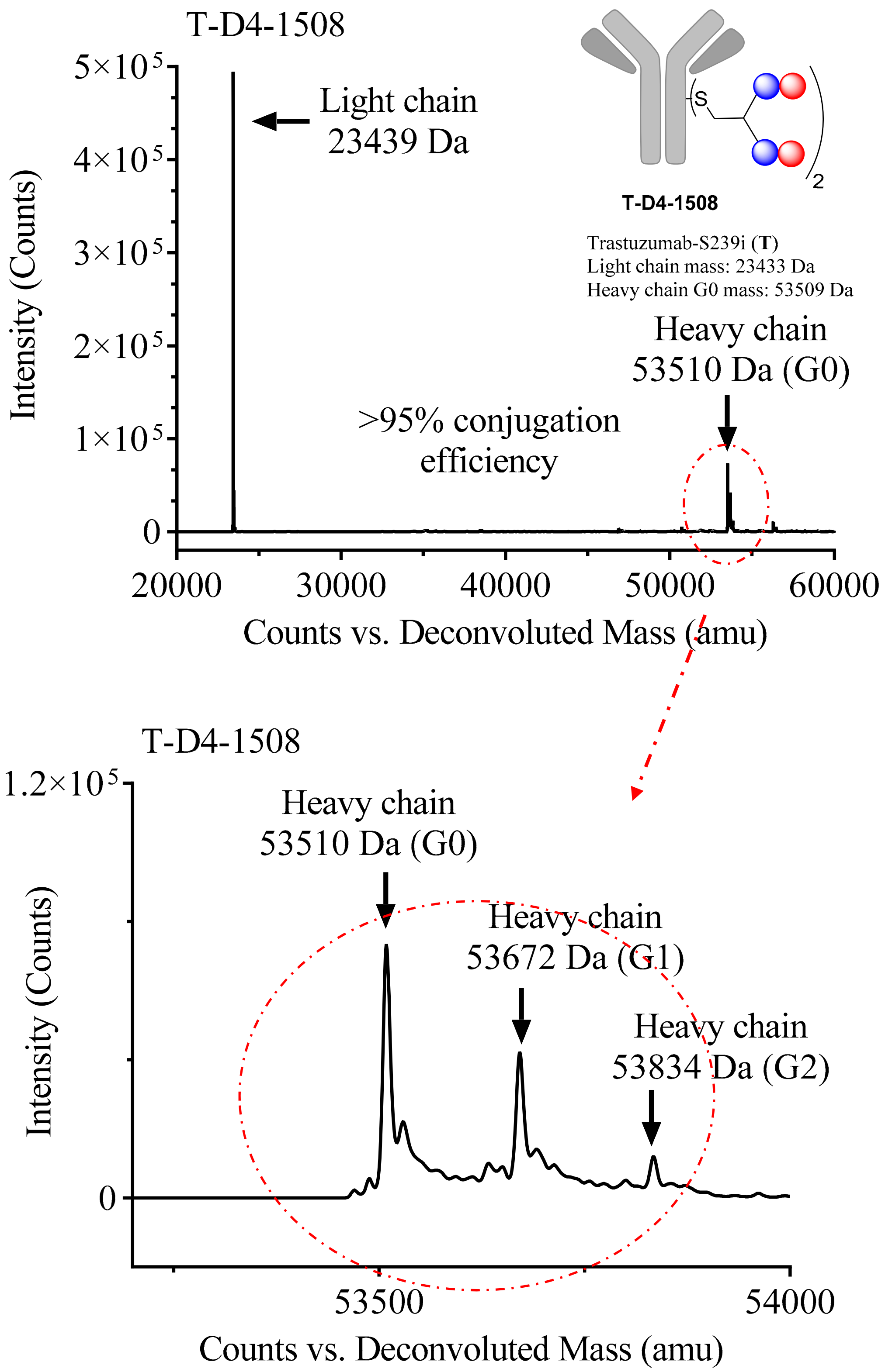
T-D4-1508
5×10^5
4×10^5
3×10^5
2×10^5
1×10^5
0
Intensity (Counts)
Light chain
23439 Da
T-D4-1508
Trastuzumab-S239i (T)
Light chain mass: 23433 Da
Heavy chain G0 mass: 53509 Da
Heavy chain
53510 Da (G0)
>95% conjugation
efficiency
20000
30000
40000
50000
60000
Counts vs. Deconvoluted Mass (amu)
T-D4-1508
1.2×10^5
0
Intensity (Counts)
Heavy chain
53510 Da (G0)
Heavy chain
53672 Da (G1)
Heavy chain
53834 Da (G2)
53500
54000
Counts vs. Deconvoluted Mass (amu)

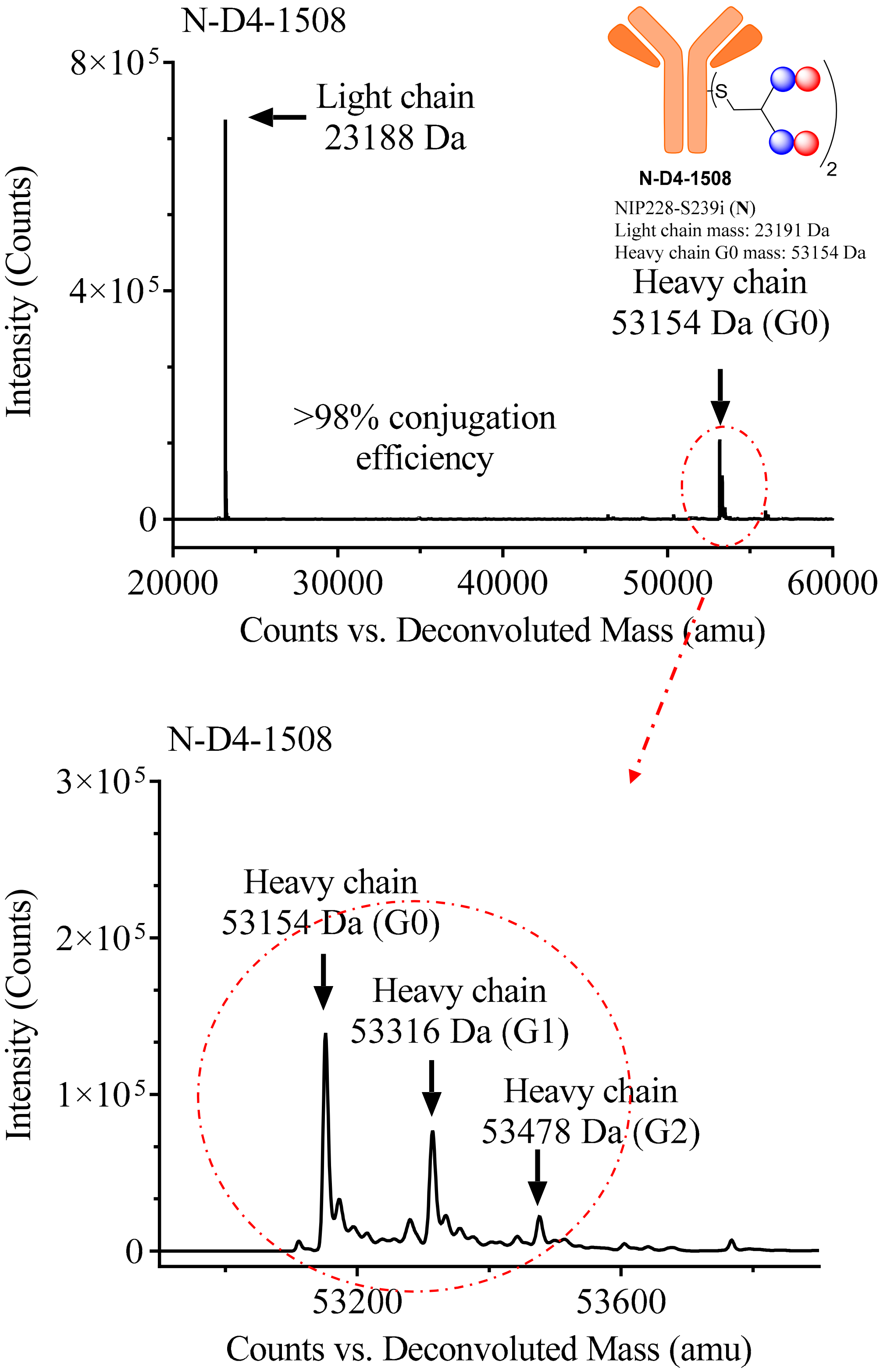
N-D4-1508
8×10^5
4×10^5
0
Intensity (Counts)
Light chain
23188 Da
N-D4-1508
NIP228-S239i (N)
Light chain mass: 23191 Da
Heavy chain G0 mass: 53154 Da
Heavy chain
53154 Da (G0)
>98% conjugation
efficiency
20000
30000
40000
50000
60000
Counts vs. Deconvoluted Mass (amu)
N-D4-1508
3×10^5
2×10^5
1×10^5
0
Intensity (Counts)
Heavy chain
53154 Da (G0)
Heavy chain
53316 Da (G1)
Heavy chain
53478 Da (G2)
53200
53600
Counts vs. Deconvoluted Mass (amu)

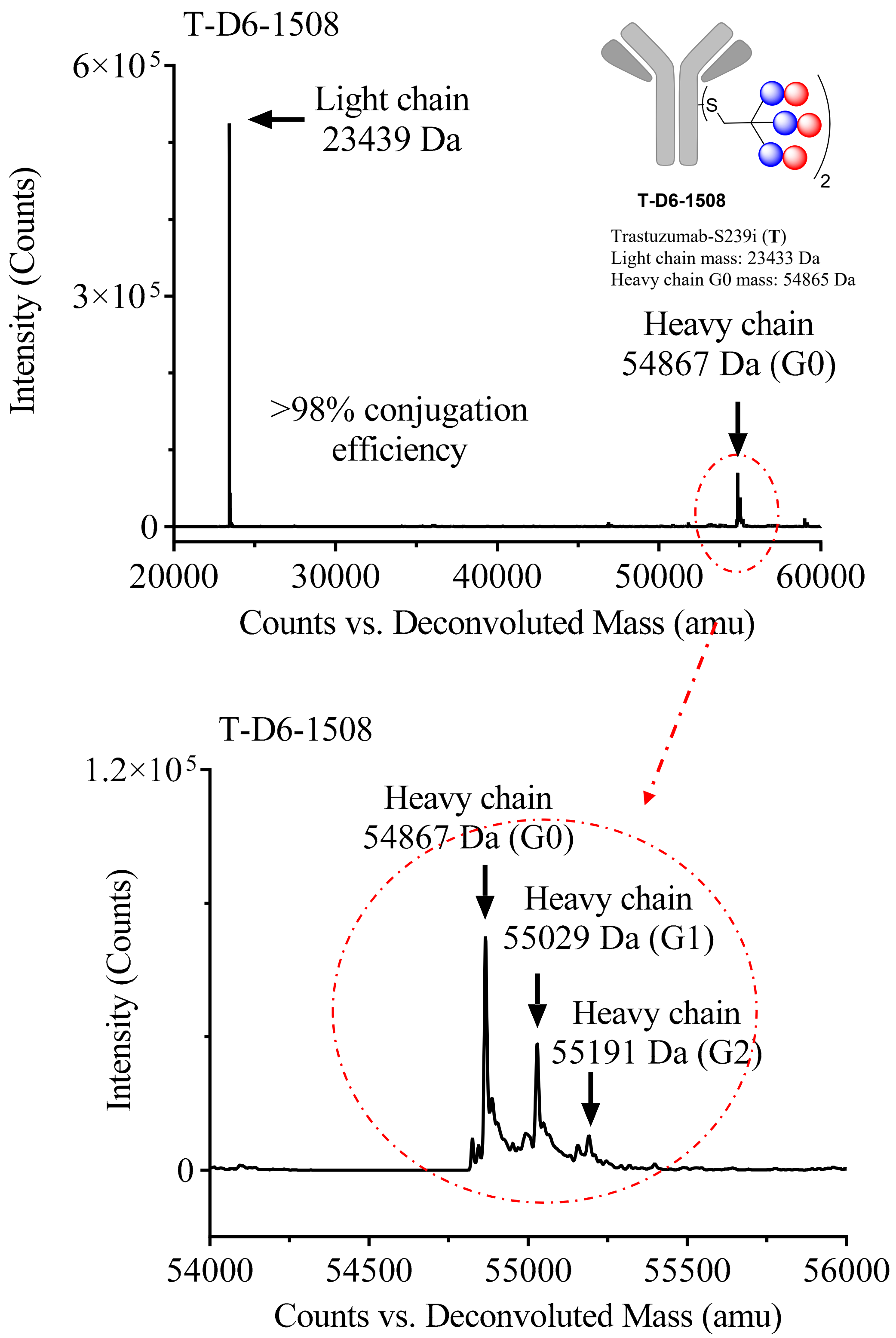
T-D6-1508
6×10^5
3×10^5
0
Intensity (Counts)
Light chain
23439 Da
>98% conjugation
efficiency
Heavy chain
54867 Da (G0)
T-D6-1508
Trastuzumab-S239i (T)
Light chain mass: 23433 Da
Heavy chain G0 mass: 54865 Da
20000
30000
40000
50000
60000
Counts vs. Deconvoluted Mass (amu)
T-D6-1508
1.2×10^5
0
Intensity (Counts)
Heavy chain
54867 Da (G0)
Heavy chain
55029 Da (G1)
Heavy chain
55191 Da (G2)
54000
54500
55000
55500
56000
Counts vs. Deconvoluted Mass (amu)

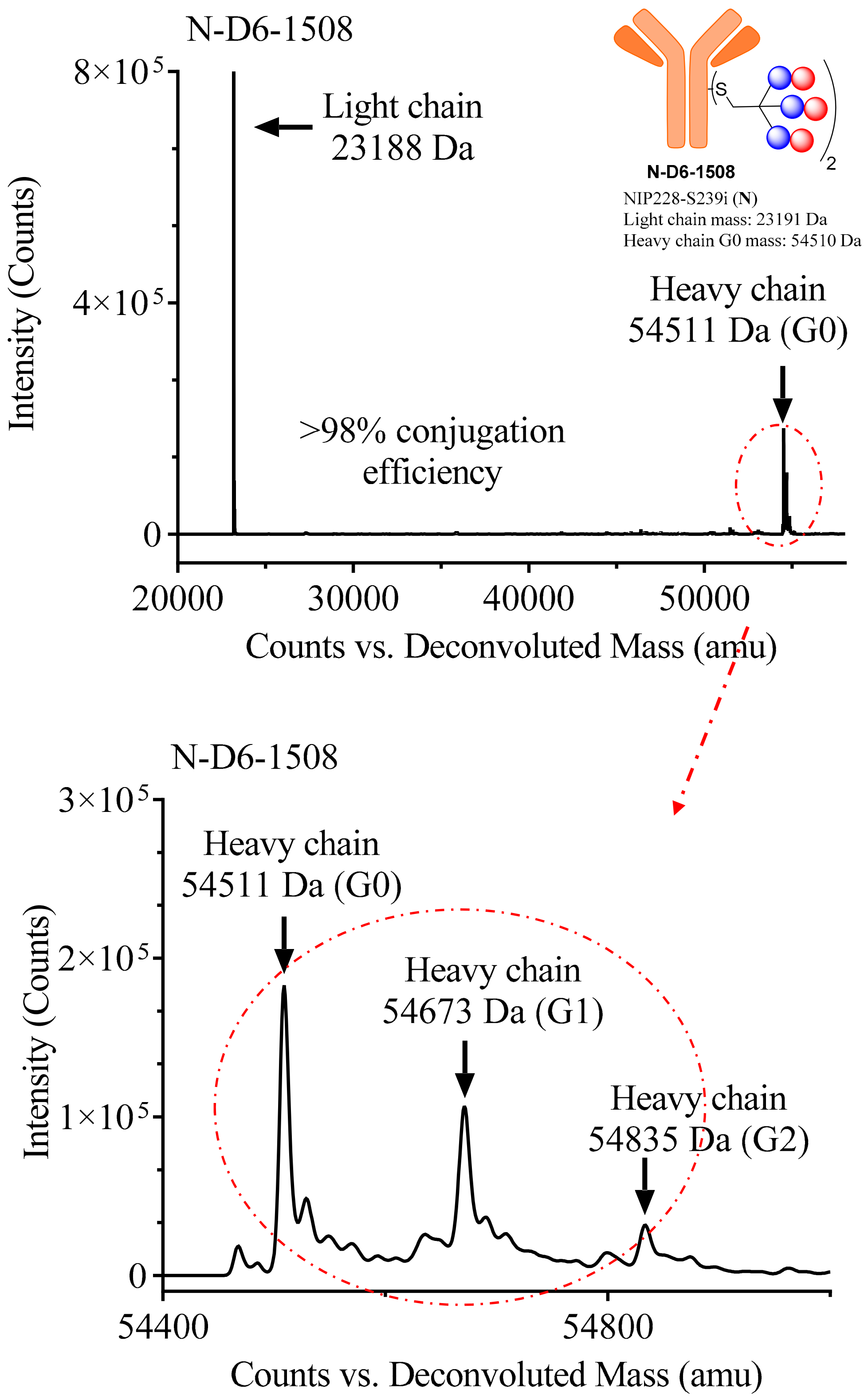
N-D6-1508
8×10^5
4×10^5
0
Intensity (Counts)
Light chain
23188 Da
>98% conjugation
efficiency
Heavy chain
54511 Da (G0)
N-D6-1508
NIP228-S239i (N)
Light chain mass: 23191 Da
Heavy chain G0 mass: 54510 Da
20000
30000
40000
50000
Counts vs. Deconvoluted Mass (amu)
N-D6-1508
3×10^5
2×10^5
1×10^5
0
Intensity (Counts)
Heavy chain
54511 Da (G0)
Heavy chain
54673 Da (G1)
Heavy chain
54835 Da (G2)
54400
54800
Counts vs. Deconvoluted Mass (amu)

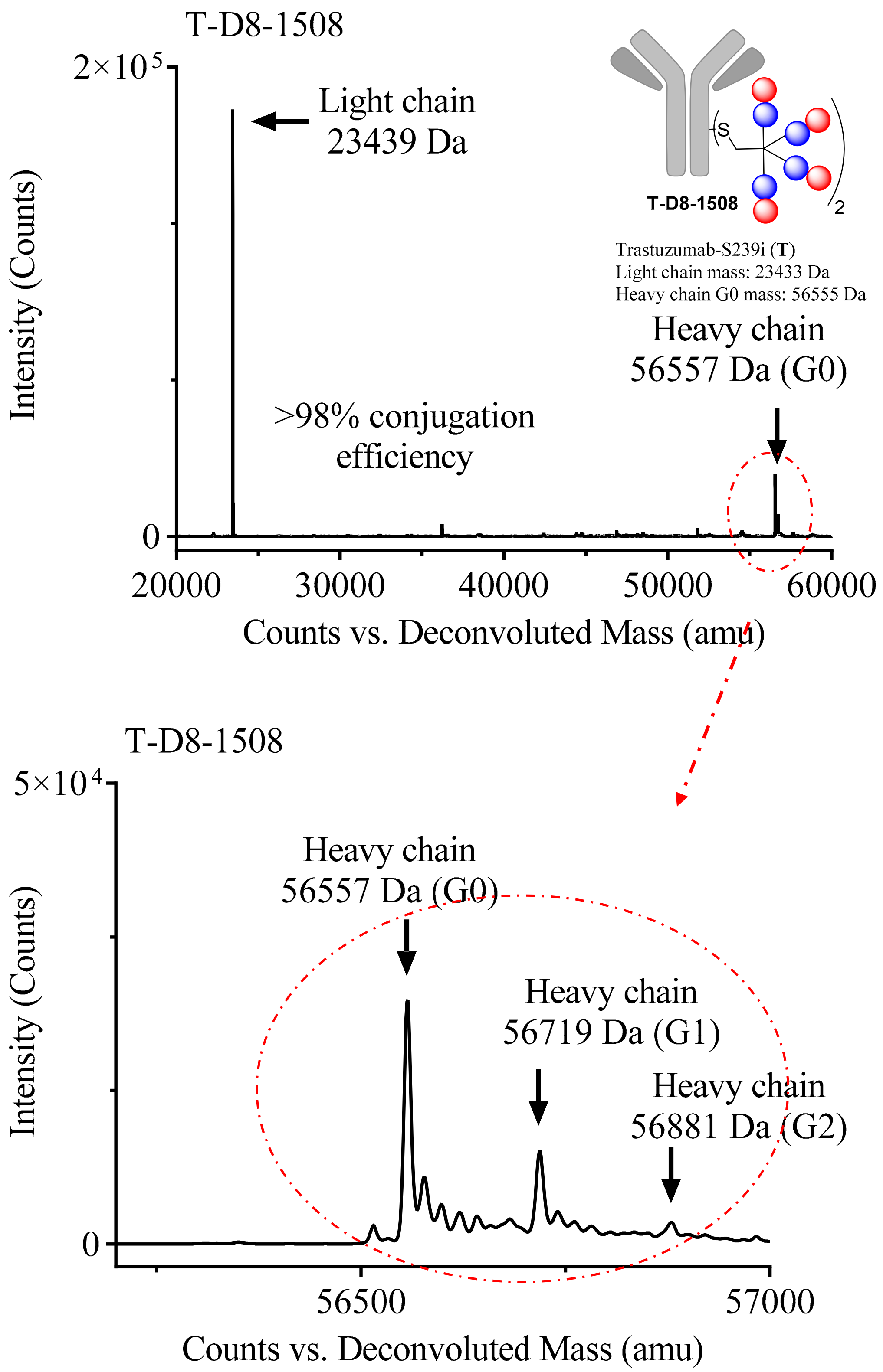
T-D8-1508
2×10^5
0
Light chain
23439 Da
T-D8-1508
Trastuzumab-S239i (T)
Light chain mass: 23433 Da
Heavy chain G0 mass: 56555 Da
Heavy chain
56557 Da (G0)
>98% conjugation
efficiency
Intensity (Counts)
20000
30000
40000
50000
60000
Counts vs. Deconvoluted Mass (amu)
T-D8-1508
5×10^4
0
Heavy chain
56557 Da (G0)
Heavy chain
56719 Da (G1)
Heavy chain
56881 Da (G2)
Intensity (Counts)
56500
57000
Counts vs. Deconvoluted Mass (amu)

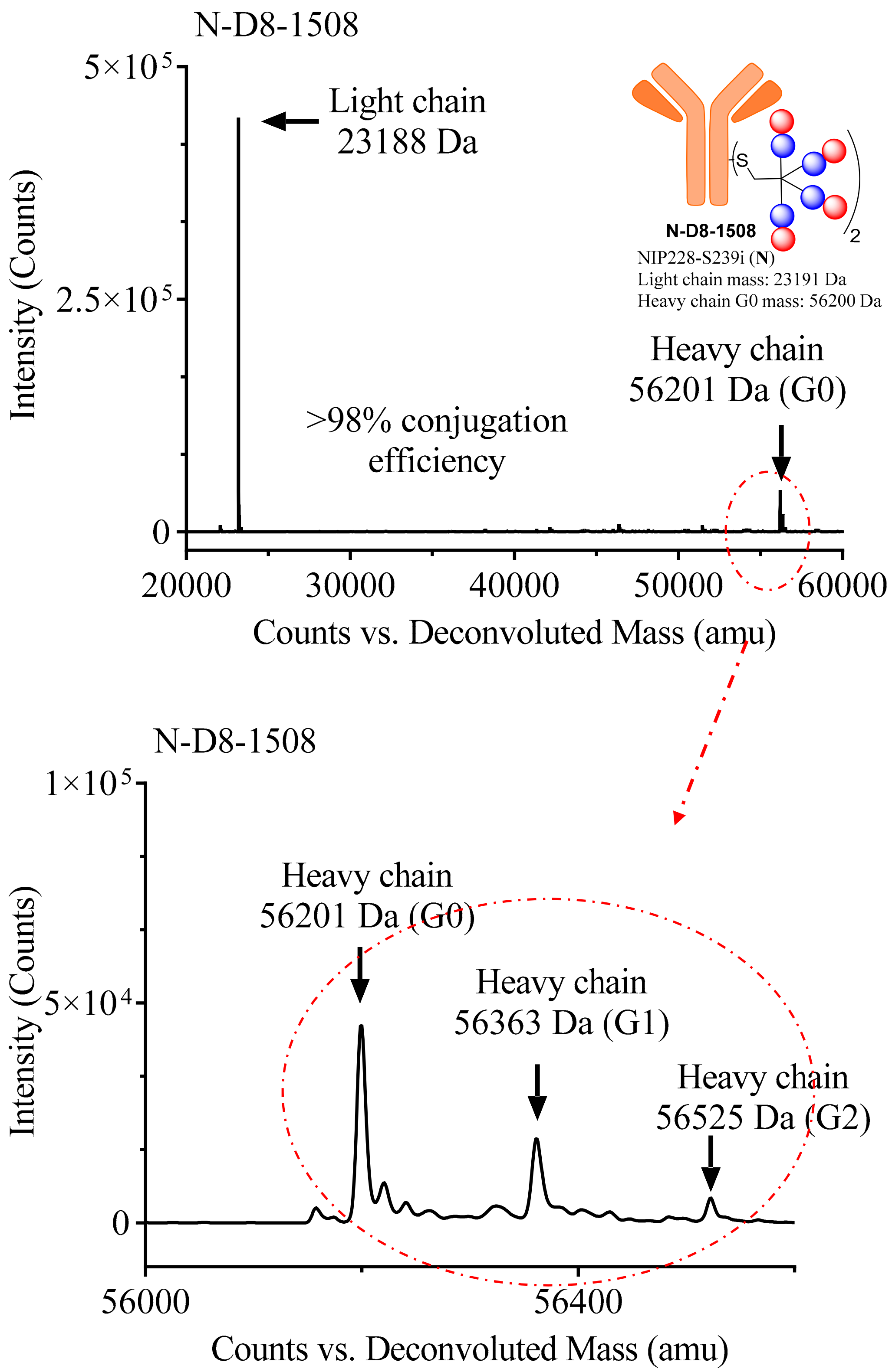

N-D8-1508
5×10⁵
2.5×10⁵
0
Intensity (Counts)
Light chain
23188 Da
N-D8-1508
NIP228-S239i (N)
Light chain mass: 23191 Da
Heavy chain G0 mass: 56200 Da
Heavy chain
56201 Da (G0)
>98% conjugation
efficiency
20000
30000
40000
50000
60000
Counts vs. Deconvoluted Mass (amu)
N-D8-1508
1×10⁵
5×10⁴
0
Intensity (Counts)
Heavy chain
56201 Da (G0)
Heavy chain
56363 Da (G1)
Heavy chain
56525 Da (G2)
56000
56400
Counts vs. Deconvoluted Mass (amu)

In vitro cytotoxicity assays (Materials)

Human breast cancer MDA-MB-361, MDA-MB-468 and gastric cancer NCI-N87 cell lines were obtained from the American Type Culture Collection (ATCC). The cells were grown in RPMI1640 (Life Technologies) supplemented with 10% heat inactivated fetal bovine serum (FBS, Life technologies) at 37°C in a humidified, 5% CO2 atmosphere. All cell lines were authenticated by short tandem repeat (STR) DNA profiling using real-time PCR analyses (IDEXX Bioresearch Laboratories).

In vitro cytotoxicity assays (Procedure)

Three tumor cell lines, MDA-MB-468, NCI-N87 and MDA-MB-361 in exponential growth phase were seeded in 96-well culture plates at 2000, 1600 and 5000 per well in 80 µl respectively, allowed to adhere overnight and treated on the following day with 20 µl of serial dilutions of ADCs in duplicate. The treated cells were cultured for another 3 days for MDA-MB-468 and 6 days for NCI-N87 and MDA-MB-361 cells. Then the cell viability was determined by the CellTiter-Glo Luminescent Viability Assay (Promega) according to manufacturer's protocol. IC50 of the ADC cytotoxicity was determined by using logistic non-linear regression analysis with Prism software (GraphPad).

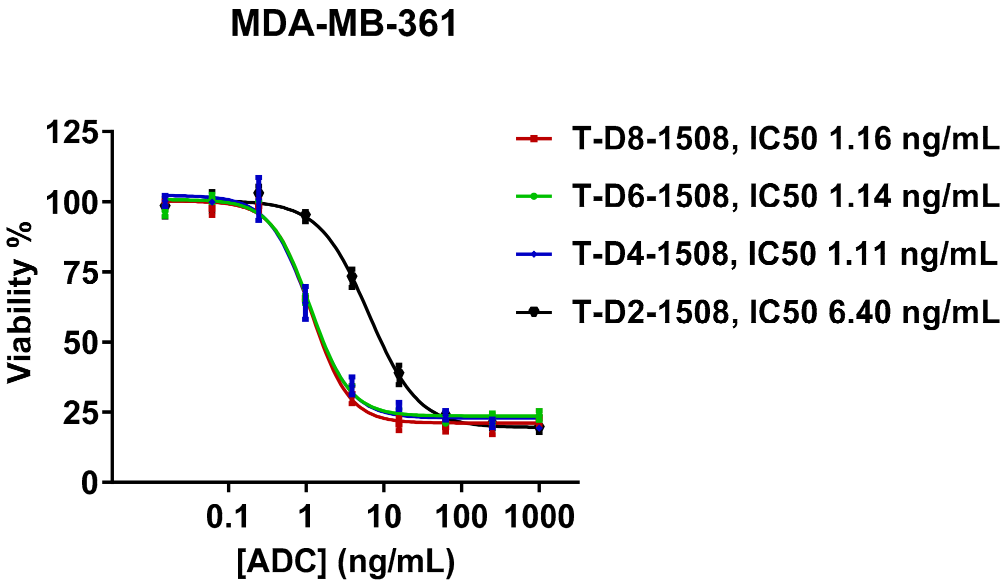

Figure S(1). In vitro cytotoxicity of Trastuzumab based ADCs obtained via hinge disulfide conjugation using Her2-expressing MDA-MB-361 breast cancer cell line.

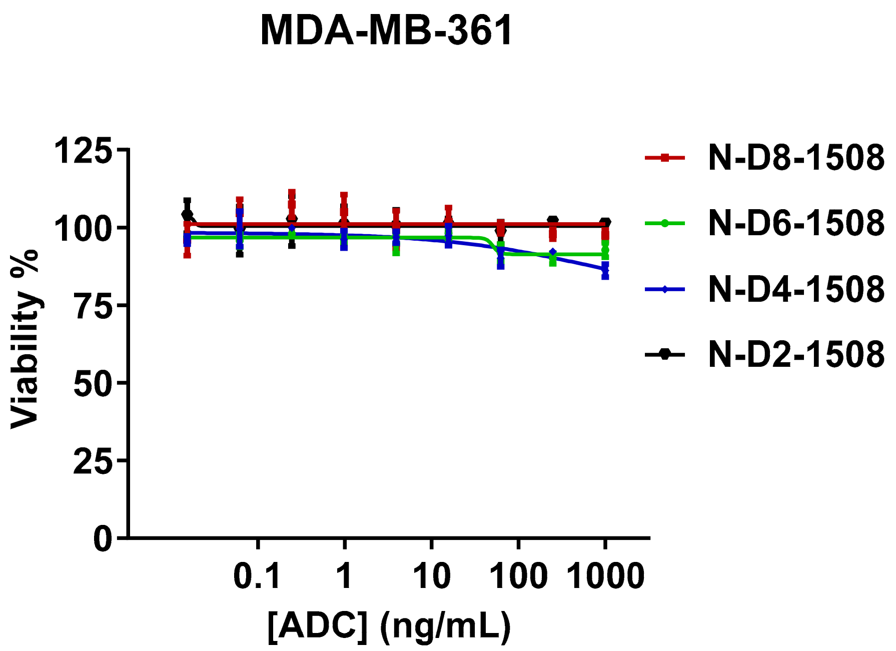

Figure S(2). In vitro cytotoxicity of NIP228 based ADCs obtained via classical cysteine conjugation using Her2-expressing MDA-MB-361 breast cancer cell line.

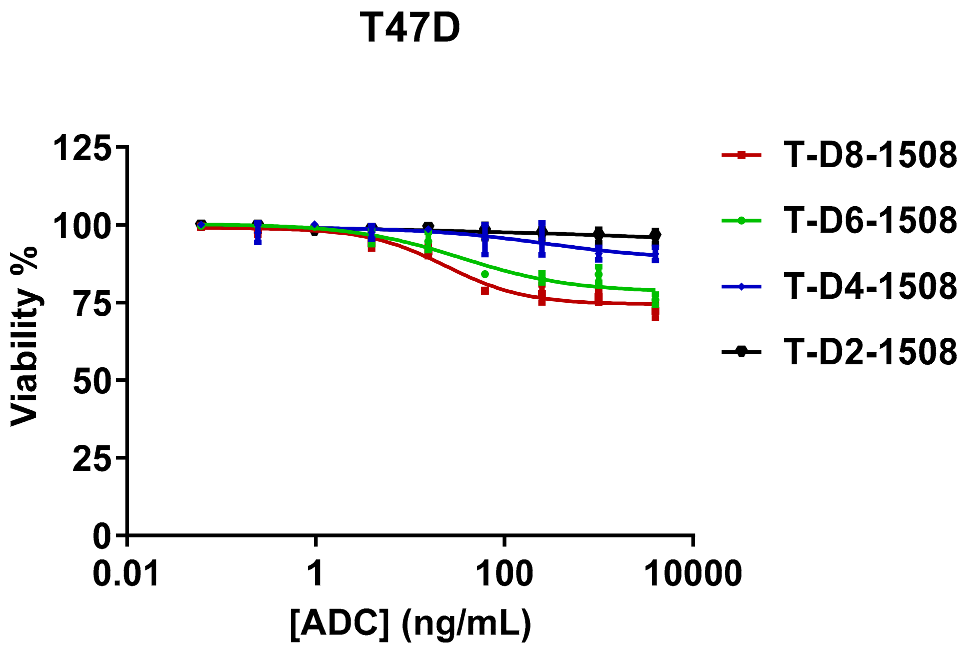

Figure S(3). In vitro cytotoxicity of Trastuzumab based ADCs obtained via hinge disulfide conjugation using Her2-expressing T47D breast cancer cell line.

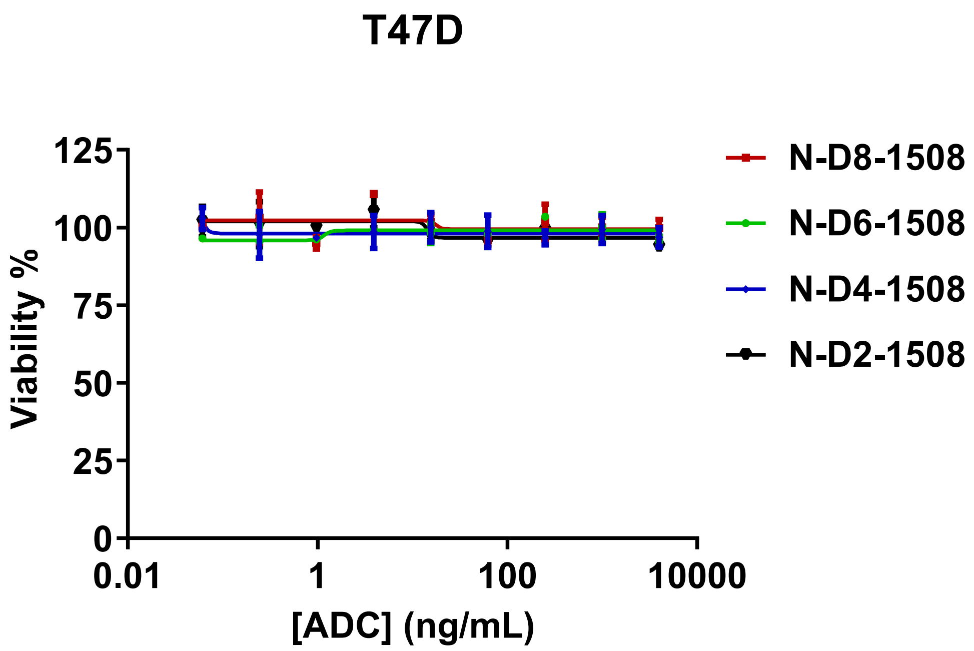

Figure S(4). In vitro cytotoxicity of NIP228 based ADCs obtained via classical cysteine conjugation using Her2-expressing T47D breast cancer cell line.

References

1. Galeone, A. et al; Bioorganic & Medicinal Chemistry Letters, 11(3), 383-386; 2001

2. A. H. St. Amant et al; Bioconjugate Chem. 2019, 30, 2340–2348

3. Gupta, Abhishek et al; New Journal of Chemistry, 41(7), 2735-2744; 2017

4. Dubowchik, Gene M. et al; U.S. Pat. Appl. Publ. (2018), US 20180222960 A1 20180809.
